# Dependency of NELF-E-SLUG-KAT2B epigenetic axis in breast cancer carcinogenesis

**DOI:** 10.1038/s41467-023-38132-1

**Published:** 2023-04-28

**Authors:** Jieqiong Zhang, Zhenhua Hu, Hwa Hwa Chung, Yun Tian, Kah Weng Lau, Zheng Ser, Yan Ting Lim, Radoslaw M. Sobota, Hwei Fen Leong, Benjamin Jieming Chen, Clarisse Jingyi Yeo, Shawn Ying Xuan Tan, Jian Kang, Dennis Eng Kiat Tan, Ieng Fong Sou, Urszula Lucja McClurg, Manikandan Lakshmanan, Thamil Selvan Vaiyapuri, Anandhkumar Raju, Esther Sook Miin Wong, Vinay Tergaonkar, Ravisankar Rajarethinam, Elina Pathak, Wai Leong Tam, Ern Yu Tan, Wee-Wei Tee

**Affiliations:** 1 Chromatin Dynamics and Disease Epigenetics Lab, Institute of Molecular and Cell Biology (IMCB), Agency for Science, Technology and Research (A*STAR), 61 Biopolis Drive, Proteos, Singapore, 138673 Republic of Singapore; 2 Department of Physiology, Yong Loo Lin School of Medicine, National University of Singapore, Singapore, 117593 Republic of Singapore; 3 Department of Oncology, Jiangsu Province Hospital of Chinese Medicine, Affiliated Hospital of Nanjing University of Chinese Medicine, 210004 Nanjing, People’s Republic of China; 4 Department of Pathology, National University Hospital, 5 Lower Kent Ridge Road, Singapore, 119074 Republic of Singapore; 5 Functional Proteomics Laboratory, SingMass National Laboratory, Institute of Molecular and Cell Biology (IMCB), Agency for Science, Technology and Research (A*STAR), 61 Biopolis Drive, Proteos, Singapore, 138673 Republic of Singapore; 6 Institute of Systems, Molecular and Integrative Biology, University of Liverpool, Liverpool, L69 7ZB UK; 7 Institute of Molecular and Cell Biology (IMCB), Agency for Science, Technology and Research (A*STAR), 61 Biopolis Drive, Proteos, Singapore, 138673 Republic of Singapore; 8 NUS Centre for Cancer Research, Yong Loo Lin School of Medicine, National University of Singapore, 14 Medical Drive, Singapore, 117599 Republic of Singapore; 9 Department of Biochemistry, Yong Loo Lin School of Medicine, National University of Singapore, 8 Medical Drive, Singapore, 117597 Republic of Singapore; 10 Advanced Molecular Pathology Laboratory, Institute of Molecular and Cell Biology, Agency for Science, Technology and Research (A*STAR), 61 Biopolis Drive, Proteos, Singapore, 138673 Republic of Singapore; 11 Genome Institute of Singapore, Agency for Science, Technology and Research (A*STAR), 60 Biopolis Drive, Genome, Singapore, 138672 Republic of Singapore; 12 Cancer Science Institute of Singapore, National University of Singapore, 14 Medical Drive, Singapore, 117599 Republic of Singapore; 13 Department of General Surgery, Tan Tock Seng Hospital, Singapore, 308433 Republic of Singapore; 14 Lee Kong Chian School of Medicine, Nanyang Technological University, Singapore, 308232 Republic of Singapore

**Keywords:** Breast cancer, Chromatin, Transcription, Epigenetics, Gene expression

## Abstract

Cancer cells undergo transcriptional reprogramming to drive tumor progression and metastasis. Using cancer cell lines and patient-derived tumor organoids, we demonstrate that loss of the negative elongation factor (NELF) complex inhibits breast cancer development through downregulating epithelial-mesenchymal transition (EMT) and stemness-associated genes. Quantitative multiplexed Rapid Immunoprecipitation Mass spectrometry of Endogenous proteins (qPLEX-RIME) further reveals a significant rewiring of NELF-E-associated chromatin partners as a function of EMT and a co-option of NELF-E with the key EMT transcription factor SLUG. Accordingly, loss of NELF-E leads to impaired SLUG binding on chromatin. Through integrative transcriptomic and genomic analyses, we identify the histone acetyltransferase, KAT2B, as a key functional target of NELF-E-SLUG. Genetic and pharmacological inactivation of KAT2B ameliorate the expression of EMT markers, phenocopying NELF ablation. Elevated expression of NELF-E and KAT2B is associated with poorer prognosis in breast cancer patients, highlighting the clinical relevance of our findings. Taken together, we uncover a crucial role of the NELF-E-SLUG-KAT2B epigenetic axis in breast cancer carcinogenesis.

## Abstract (web summary)

Transcriptional dysregulation contributes to tumor progression. Here the authors show that transcriptional complex NELF interacts with SLUG, and co-opts KAT2B, to promote the expression of epithelial-mesenchymal transition (EMT) and stemness-associated genes in breast cancer.

## Introduction

The dynamic nature of transcription regulation provides flexibility in gene expression, which is vital for the faithful determination of cell identity and proper development of multicellular organisms^1^. However, cancer cells can exploit this inherent transcriptional plasticity to drive opportunistic adaption to the tumor microenvironment. In addition to genetic mutations and copy number alterations, transcriptional and epigenetic variations have been shown to contribute to cancer cell plasticity, resulting in enhanced survival fitness and resistance to therapeutic pressures. Given the relative challenge of correcting genetic aberrations, the reversibility and context-specific nature of epigenetic and transcriptional changes offer promising therapeutic opportunities^2,3^.

Transcriptional regulators have been thought to perform vital homeostatic functions in all tissues, inhibition of which may result in the indiscriminate killing of normal cells. However, several studies have demonstrated that many transcriptional regulators can in fact exert context-specific effects in healthy tissues, and can be further expropriated in malignant cells to drive selective oncogenic gene expression programs, leading to transcriptional dysregulation that contributes to cancer^4–8^. For example, oncogenic genes such as *c-MYC* display a disproportionate response to transcriptional inhibition, resulting in the preferential killing of malignant cells^9,10^. This has given rise to the concept of ‘transcription addiction’ in cancer cells^3^. As such, transcription-based therapies that target different aspects of the transcription cycle, from transcription initiation to elongation, have gained traction in the last few years^11^. Of note, a recent study identified transcription initiation and elongation factors as important players of in vivo cancer dependencies, emphasizing how dysregulated transcription can give rise to tumorigenesis that can be harnessed for the development of new therapeutic candidates^12^. Targeting cancer cell transcriptional dependency may therefore be an attractive therapeutic strategy for multiple cancers, beyond existing treatments that target oncogenic signaling and immune-related pathways.

NELF is a four-subunit transcriptional complex that consists of NELF-A, B, C/D, and E^13–16^. The physiological importance of NELF has been well described in different biological contexts, ranging from playing an essential function in early mouse embryonic development to modulating stress and inflammatory responses in human cells^17–23^. These studies highlighted the role of NELF in fine-tuning transcriptional outputs in response to cellular and external cues, thereby positioning it at the nexus between transcription and signaling response. However, the mechanistic basis by which NELF exerts its gene regulatory functions remains to be fully elucidated. For example, whereas in vitro biochemical studies have unequivocally established a role of NELF in promoting RNA Polymerase II (RNAPII) pausing at the proximal-promoter region of target genes, which is also observed on select loci in vivo^21^, other reports showed that disruption of NELF function in cells led a general decrease in RNAPII occupancy at both promoters and gene bodies^23–32^. Recently, it was found that acute depletion of NELF did not lead to a global release of RNAPII but may affect other promoter-proximal regulatory steps such as 5’ mRNA cap stability^30^. Taken together, these results highlight a more complex scenario in which the gene regulatory functions of NELF likely depend on the chromatin context and its interaction with other partner proteins in vivo.

Many studies have demonstrated that cancer cells co-opt the core transcription machinery to sustain their oncogenic state. Notable examples include BRD4 and CDK7, which have emerged as promising therapeutic targets in cancer^10,33^. Comparatively, the role of NELF in cancer is not fully understood. Considering its function as a molecular rheostat that coordinates transcription with environmental inputs^34^, we hypothesized that NELF may also participate in cancer cell adaptive transcriptional responses downstream of oncogenic driver events. Thus, targeting NELF may serve as an attractive therapeutic strategy to attenuate oncogenic transcriptional programs for impeding cancer progression.

Here, we show that disruption of the NELF complex inhibits tumorigenesis and metastasis of multiple breast cancer cell lines of different molecular subtypes. We uncover the ability of NELF to promote the expression of epithelial-mesenchymal transition (EMT) and stemness-associated genes, which are implicated in cancer metastasis and disease recurrence. Specifically, we show that NELF-E, a key subunit of NELF, interacts with the EMT transcription factor (TF), SLUG, to promote the transcription of key EMT target genes. Integration of genomic and transcriptomic data led us to identify the histone acetyltransferase, KAT2B, as a key transcriptional target of NELF-E-SLUG. Notably, genetic and pharmacological inactivation of KAT2B results in impaired EMT and loss of stemness properties, similar to NELF-E ablation, thereby establishing the functional importance of the NELF-E-KAT2B regulatory axis in breast cancer carcinogenesis.

## Results

### Loss of NELF leads to impaired tumorigenesis in vitro and in vivo

**Fig. 1:**
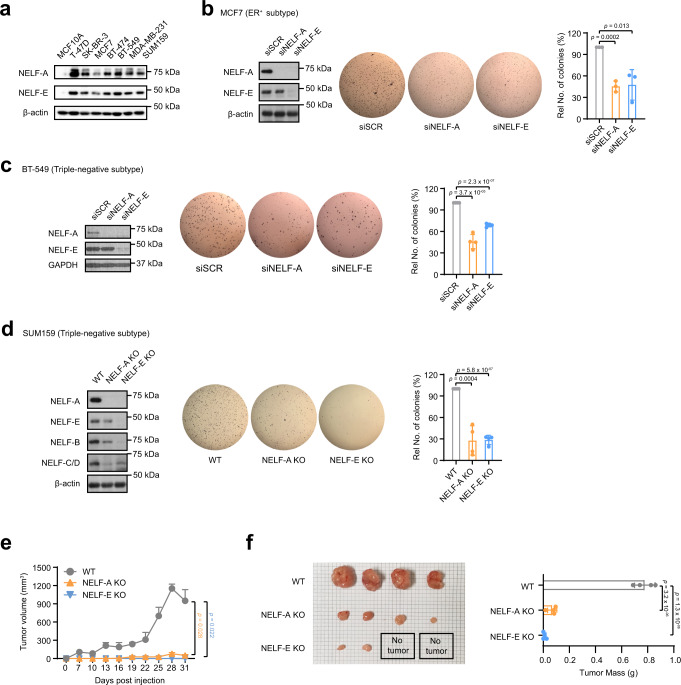
Abolishment of the NELF complex impairs tumorigenic properties. **a** Western blot analysis of NELF-A and NELF-E in non-tumorigenic breast epithelial cell line MCF10A, as well as in different breast cancer cell lines T-47D (ER^+^, PR^+^), SK-BR-3 (HER2^+^), MCF7 (ER^+^), BT-474 (HER2^+^), BT-549 (triple-negative), MDA-MB-231 (triple-negative), and SUM159 (triple-negative). β-actin was used as the loading control. **b**, **c** Left: MCF7 cells (*n* = 3) and BT-549 cells (*n* = 4) were transfected with non-targeting siRNA (‘scrambled, siSCR’) and siRNAs targeting NELF-A and NELF-E, respectively, followed by western blot analysis. GAPDH was used as the loading control. Right: Representative images and quantification of soft agar assays. **d** Left: Western blot analysis of NELF-A, NELF-E, NELF-B, and NELF-C/D in WT and NELF-A/NELF-E KO SUM159 cells. β-actin was used as the loading control. Right: Representative images and quantification of soft agar assays (*n* = 4). **e** 5 × 10^6^ WT, NELF-A KO, and NELF-E KO SUM159 cells were injected into the mammary fat pads of NOD/SCID mice, respectively. Tumor volume was measured every 3 days after the tumor was palpable; mean ± SEM, *n* = 4/group. **f** Left: Tumors harvested from WT, NELF-A KO, and NELF-E KO groups; Right: Quantification of tumor weights; mean ± SEM, *n* = 4/group. Blots and images are representative of at least three independent experiments. Data in (**b**–**d**) are presented as mean ± SD. *p*-values are determined by a two-tailed Student’s *t*-test. Source data are provided as a Source Data file.

The four NELF subunits are interdependent for their protein stability, wherein the loss of any one subunit can affect the stability of others. In particular, NELF-A and NELF-E are two critical functional subunits of the NELF complex that bind to RNAPII and nascent RNA, respectively^13,15,16^, and may further exist as a subcomplex in vivo^35^. To establish if the NELF complex plays a role in tumorigenesis, we decided to focus on these two critical subunits in the first instance. First, we examined the expression of NELF-A and NELF-E in different breast cancer cell lines and found that both were highly upregulated in all the breast cancer cell lines tested, compared to the non-tumorigenic human mammary epithelial cell lines MCF10A and HMEC (Fig. 1a, Supplementary Fig. 1a). Next, we depleted NELF-A and NELF-E separately and assessed the impact on anchorage-independent growth in different breast cancer cell lines. Specifically, siRNA/shRNA-mediated knockdown (KD) of NELF-A or NELF-E in breast cancer cell lines of different subtypes (ER^+^, HER2^+^, and triple-negative) led to reduced anchorage-independent growth in vitro (Fig. 1b, c and Supplementary Fig. 1b, c). In contrast, no appreciable decrease in cell viability was observed in non-tumorigenic MCF10A and HMEC cell lines following NELF-E KD (Supplementary Fig. 1d), highlighting a greater dependency for NELF in breast cancer cells.

To study the tumorigenic effect of NELF in vivo, we generated CRISPR-mediated genetic knockouts (KOs) of NELF-A and NELF-E in the highly aggressive SUM159 breast cancer cell line (triple-negative subtype). We first confirmed that loss of NELF-A or NELF-E led to reduced protein expression of other NELF subunits, in agreement with previous reports^23,30^ (Fig. 1d). Similar to the NELF KD breast cancer cell lines described above, NELF-A and NELF-E KO SUM159 cells also showed decreased colony formation in soft agar assays (Fig. 1d). Next, we transplanted wild type (WT), NELF-A KO, and NELF-E KO SUM159 cells into the mammary fat pads of NOD/SCID mice to evaluate the tumorigenic potential of these cells in vivo. In this xenograft study, we observed a significant reduction in tumor growth for both NELF-A and NELF-E KO cells compared to the WT cells (Fig. 1e, f). Taken together, our in vitro and in vivo results demonstrated a crucial role of the NELF complex in breast cancer tumorigenesis.

### EMT and stemness-associated pathways are downregulated in NELF-depleted cells

**Fig. 2:**
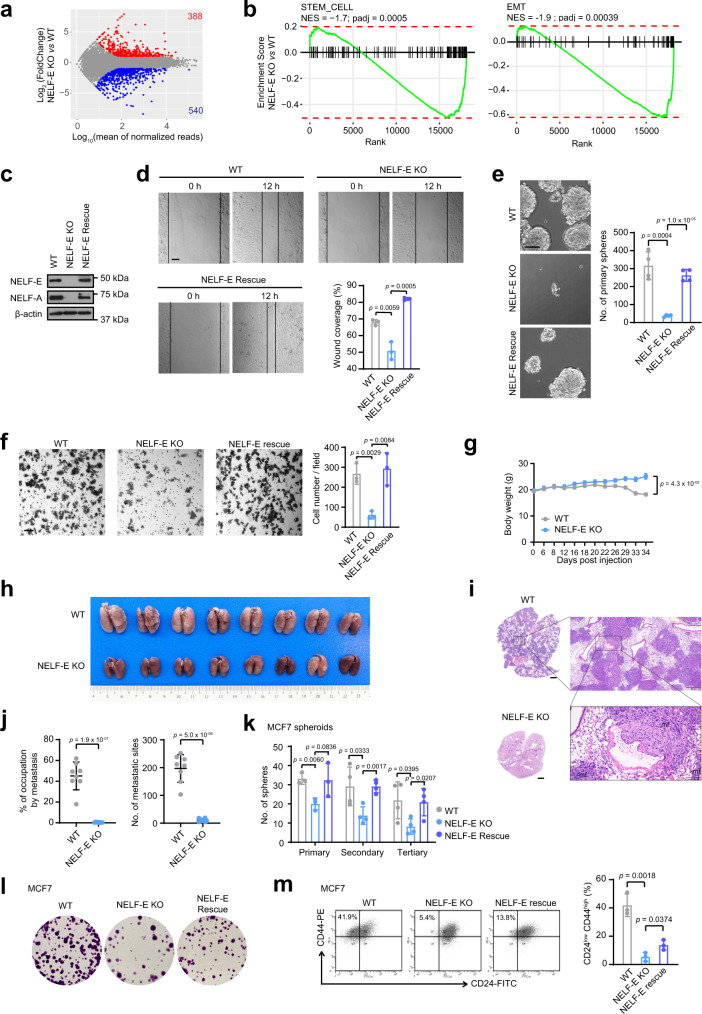
NELF depletion leads to reduced stemness-like traits and downregulated EMT pathway. **a** MA plot showing gene expression changes between NELF-E KO and WT SUM159 cells. Red and blue points indicate significantly upregulated (*n* = 388) and downregulated (*n* = 540) genes, respectively. **b** GSEA enrichment plot for stemness and epithelial-mesenchymal transition pathways in NELF-E KO SUM159 cells. **c** Western blot analysis of NELF-E and NELF-A in WT, NELF-E KO, and NELF-E rescue SUM159 cells. β-actin was used as the loading control. **d** Quantification of wound healing assay in WT, NELF-E KO, and NELF-E rescue SUM159 cells. Scale bar = 100 μm. **e** Quantification of mammosphere formation assay in WT, NELF-E KO, and NELF-E rescue SUM159 cells (*n* = 4). Scale bar = 100 μm. **f** Quantification of invasion assay in WT, NELF-E KO, and NELF-E rescue SUM159 cells (*n* = 3). Scale bar = 100 μm. **g** Body weight (mean ± SEM) of mice injected with 1.25 × 10^6^ WT and NELF-E KO SUM159 cells, respectively (*n* = 8, each group). **h** Lungs inflated and fixed with 10% neutral buffer formalin from mice in WT (*n* = 8) and NELF-E KO group (*n* = 8). **i** Representative images of H&E staining in lung tissues at three magnifications (mf: metastatic foci). From top to bottom, Bar = 2 mm, 500 μm, and 100 μm, respectively. **j** Quantitative analysis of lung metastasis presented as % of occupation by metastasis and the number of metastatic sites per lung (*n* = 8/group). **k** Graph showing quantification of mammospheres in WT, NELF-E KO, and NELF-E rescue MCF7 cells at primary (*n* = 3), secondary (*n* = 4), and tertiary passages (*n* = 4). **l** MCF7 cells from tertiary spheroids were seeded at a density of 1000 cells per well and incubated for 2 weeks. The colonies were then fixed and stained with crystal violet. **m** Flow cytometry analysis and quantification of the CD24^low^/CD44^high^ population in MCF7 tertiary mammospheres (*n* = 3). Blots and images are representative of at least three independent experiments. Data in (**d**, **e**, **f**, **j**, **k**, **m**) are presented as mean ± SD. *p*-values are determined by a two-tailed Student’s *t*-test. Source data are provided as a Source Data file.

To gain mechanistic insights into how the NELF complex regulates malignant transformation, we performed transcriptomic analyses. We focused on NELF-E KO SUM159 cells, which exhibited a pronounced impact on NELF complex integrity, showing a near complete loss of all NELF subunits (Fig. 1d). RNA sequencing (RNA-seq) of NELF-E KO cells identified 388 upregulated (UP) and 540 downregulated (DOWN) genes compared to WT cells (Fig. 2a). Gene set enrichment analysis (GSEA) from the Molecular Signatures Database (MSigDB)^36,37^ identified several pro-tumorigenic pathways to be significantly downregulated in NELF-E KO SUM159 cells (Supplementary Data 1), including the epithelial-mesenchymal transition (EMT) and mammary stem cell pathways (Fig. 2b), which we further confirmed by qPCR of select EMT and stemness-related genes (Supplementary Fig. 2a). Importantly, KD of NELF-E in another TNBC cell line, BT-549, also led to the repression of EMT pathway, highlighting a conserved effect (Supplementary Fig. 2b).

To validate the functional impact of NELF depletion on EMT and stemness properties, we performed cell migration and mammosphere-forming assays. Further to NELF-E KO SUM159 cells, we also generated a NELF-E KO rescue cell line. We confirmed that loss of NELF-E impeded cell migration and mammosphere-forming capacity, and importantly, both properties were restored in the rescue cell line (Fig. 2c–f). To evaluate the effect of NELF-E depletion on cancer metastasis in vivo, we performed tail vein injection of SUM159 WT and NELF-E KO cells in NSG mice. Compared to the NELF-E KO group, mice in the WT group exhibited body weight loss during the experimental period (Fig. 2g). More importantly, in the WT group, the lungs exhibited an extensive presence of metastasis (both the number of metastatic sites and their area of occupancy) as compared to the NELF-E KO group in the pulmonary parenchyma (Fig. 2h–j). Thus, loss of NELF led to attenuated oncogenic pathways, resulting in impaired metastasis in vivo.

Breast cancer is a highly complex disease that has been classified into several subtypes. To assess the generality of our findings beyond triple-negative breast cancer (TNBC) cells, we also generated NELF-E KO and rescue cells in an ER^+^ cell line, MCF7 (Supplementary Fig. 2c). We first validated that MCF7 NELF-E KO cells showed diminished anchorage-independent growth, which was restored in the rescue cells (Supplementary Fig. 2c). Importantly, GSEA analysis likewise identified the mammary stem cell gene signature to be significantly downregulated in MCF7 NELF-E KO cells (Supplementary Fig. 2d), which was consistent with our earlier findings in SUM159 (Fig. 2b). Serial passaging of primary mammospheres confirmed that the self-renewal capacity of MCF7 NELF-E KO cells was impaired, but recovered in the rescue cells (Fig. 2k and Supplementary Fig. 2e). We also used cells from the tertiary spheres for additional colony formation assays, which gave a similar outcome (Fig. 2l). To address if the reduced self-renewal capacity of MCF7 NELF-E KO cells may be due to a loss of cancer stem cells (CSCs) or tumor-initiating cells (TICs), we performed flow cytometry analysis using CD44 and CD24 surface markers. Consistent with our expectation, we found that the CD24^low^/CD44^high^ CSC-like population was decreased from 41.9% in WT MCF7 spheroids to 5.4% in NELF-E KO spheroids, and partially restored to 13.8% in NELF-E rescue spheroids (Fig. 2m and Supplementary Fig. 2f). Similar results were observed in adherent MCF7, T-47D, and SUM159 cells (Supplementary Fig. 2g–i). Altogether, our data in multiple breast cancer cell lines of different subtypes demonstrated a crucial role of NELF in promoting breast cancer tumorigenesis through the activation of EMT and stemness traits.

To evaluate the clinical relevance of our findings, we examined the expression of NELF-E and its relation to clinicopathological characteristics using the METABRIC and SCAN-B breast cancer datasets^38,39^. Consistent with our experimental findings, we observed that the expression of NELF-E is positively correlated with tumor grade: NELF-E expression is highest in grade 3 tumors compared to grade 1 and 2 tumors (Supplementary Fig. 3a). In addition, we noticed that higher NELF-E expression is associated with poorer overall patient survival in both METABRIC and SCAN-B cohorts, notably in the ER^+^/HER2^−^ and ER^−^/HER2^−^ subtypes (Supplementary Fig. 3b). Multivariate analysis, after controlling for ER and HER status, also revealed a significant negative correlation between NELF-E expression and patient survival in both the METABRIC and SCAN-B cohorts (METABRIC: HR = 1.13, *p* = 3.45 × 10^−^^06^; SCAN-B: HR = 1.67, *p* = 2.15 × 10^−^^04^), indicating that higher NELF-E expression is generally associated with poorer overall patient survival. Additionally, we analyzed NELF-E expression by immunohistochemistry on commercial breast cancer tissue microarrays and confirmed a higher protein expression of NELF-E in tumor tissues (grade 1 to 3) compared to normal breast tissue (grade 0) (Supplementary Fig. 3c). Collectively, these independent datasets suggest a utility of NELF-E as a putative biomarker for breast cancer, in which high expression of NELF-E may correlate with increased stem-like features, leading to greater tumor aggressiveness and poorer clinical outcome.

### Loss of NELF inhibits EMT in an inducible metastatic breast cancer cell line

**Fig. 3:**
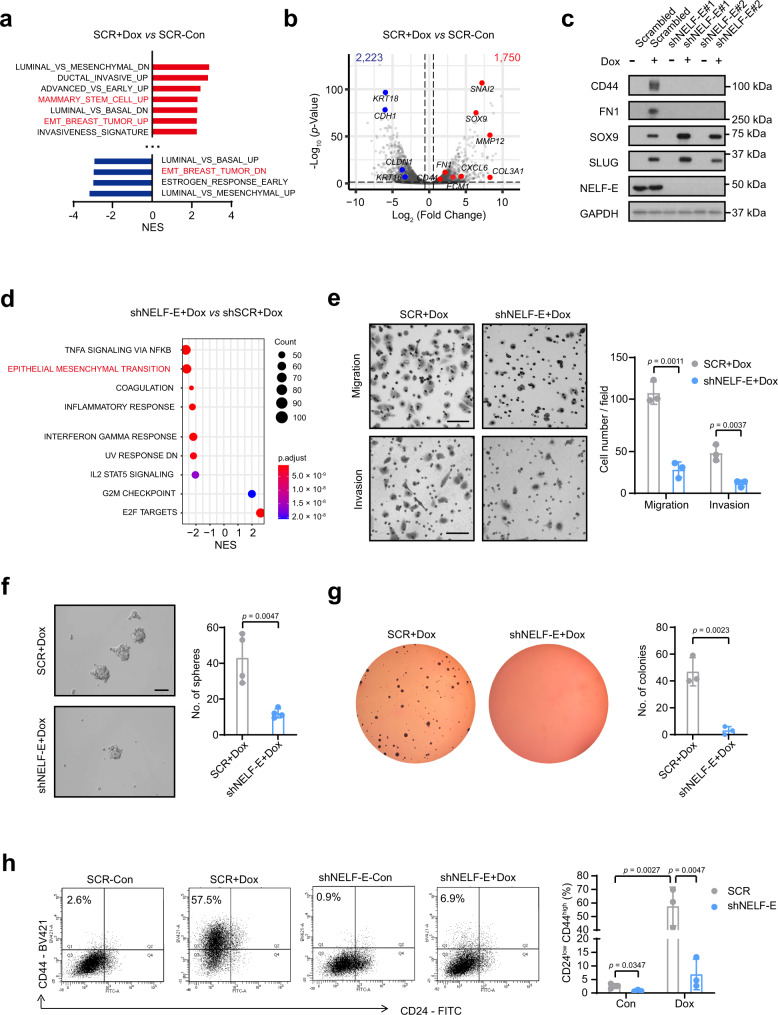
Loss of NELF-E suppresses EMT in a SOX9-SLUG overexpression MCF7 cell line model. **a** Barplot showing the top perturbed pathways from Molecular Signatures Database (MSigDB) in Doxycycline (Dox)-treated MCF7ras+SS cells compared to the vehicle control. **b** Volcano plot depicting the significantly upregulated genes (*n* = 1750) and downregulated genes (*n* = 2223). Select mesenchymal/stemness-related genes are labeled in red, while epithelial genes are labeled in blue. adj. *p*-value ≤ 0.05; |fold change| ≥ 1.5. *p*-value was calculated using DESeq2 package (see details in “Methods”) **c** Western blot analysis for different markers in Dox-induced MCF7ras+SS cells transduced with scrambled or two independent shRNAs targeting NELF-E. GAPDH was used as the loading control. **d** GSEA plot of shNELF-E+Dox vs SCR+Dox cells. *p*-value was calculated from gene set enrichment analysis. (see detail in “Methods”). **e** Representative images and quantification of the migration and invasion assays. MCF7ras+SS cells were transduced with scrambled or NELF-E shRNA and treated with Dox to induce SOX9 and SLUG expression for 72 h prior to the assays (*n* = 3). Scale Bar = 100 μm. **f** Representative images and quantification of the sphere-formation assay. MCF7ras+SS cells were transduced with scrambled or NELF-E shRNA, followed by 72-h Dox treatment. Cells were seeded at a density of 10K per well, and the number of spheres was counted 5–7 days later (*n* = 4). Scale Bar = 100 μm. **g** Representative images and quantification of the soft agar assays for SCR+Dox and shNELF-E+Dox cells (*n* = 3). **h** Flow cytometry analysis and quantification of the CD24^low^/CD44^high^ population in NELF-E KD+Dox cells compared to SCR+Dox cells (*n* = 3). Blots and images are representative of at least three independent experiments. *p*-values in (**e**–**h**) are determined by a two-tailed Student’s *t*-test. Mean ± SD is represented by bar graphs. Source data are provided as a Source Data file.

To better examine the requirement of NELF-E during EMT induction and progression, as well as its interplay with EMT transcription factors (TFs), we leveraged the doxycycline (Dox)-inducible SLUG and SOX9 MCF7ras breast cancer cell line (henceforth referred to as MCF7ras+SS)^40,41^. SLUG (or SNAI2) and SOX9 are two key TFs that regulate stemness in mammary cells, and they promote the tumorigenic and metastasis-seeding abilities of human breast cancer cells^40^. Using this cell line, we first verified that overexpression of SLUG and SOX9 activated the EMT and stem cell programs (Fig. 3a). In addition to SLUG and SOX9, notable CSC markers (such as *CD44*) and mesenchymal markers (such as *FN1* and *CXCL6*) were significantly upregulated, whereas epithelial markers (such as *CDH1* and *KRT18*) were distinctively downregulated (Fig. 3b, Supplementary Fig. 4a), recapitulating the key molecular cornerstones of EMT.

We proceeded to deplete NELF-E using two independent shRNAs (shNELF-E#1 and shNELF-E#2) in MCF7ras+SS cells and showed that FN1 and CD44 protein expressions were dramatically reduced in both cases (Fig. 3c). RNA-seq confirmed that EMT and related pathways (such as NFκB and IL2-STAT5) were downregulated in shNELF-E+Dox compared to SCR (scrambled)+Dox cells (Fig. 3d and Supplementary Fig. 4b). Likewise, the stemness pathway also showed a negative enrichment in shNELF-E+Dox cells (Supplementary Fig. 4b). We validated by qPCR that key mesenchymal and stemness-related genes were downregulated upon NELF-E KD (Supplementary Fig. 4c). We also detected a cohort of genes that are consistently downregulated upon NELF-E depletion in three different cell lines (Observed to expected ratio = 5.77, *p*-value = 5.05 × 10^−^^52^; SuperExactTest^42^) (Supplementary Fig. 4d, Top), and in particular, the EMT and stemness pathways (Supplementary Fig. 4d, Bottom). One representative example is *CAPG* (Supplementary Data 2), which is associated with poor patient prognosis^43^.

We further performed tumorigenesis assays to assess changes in cell migration, self-renewal, and anchorage-independent growth. In all cases examined, NELF-E KD led to impaired EMT and stemness properties (Fig. 3e–g), consistent with our previous findings (Fig. 2). As a case in point, in contrast to the induction of SOX9 and SLUG that led to a massive increase in CD24^low^/CD44^high^ population (57.5%), this effect was severely attenuated in NELF-E KD cells (6.9%) (Fig. 3h). Collectively, these data suggest that the EMT-inducing activities mediated by SLUG and SOX9 are dependent on NELF.

### Loss of NELF predominantly affects upregulated genes during EMT

**Fig. 4:**
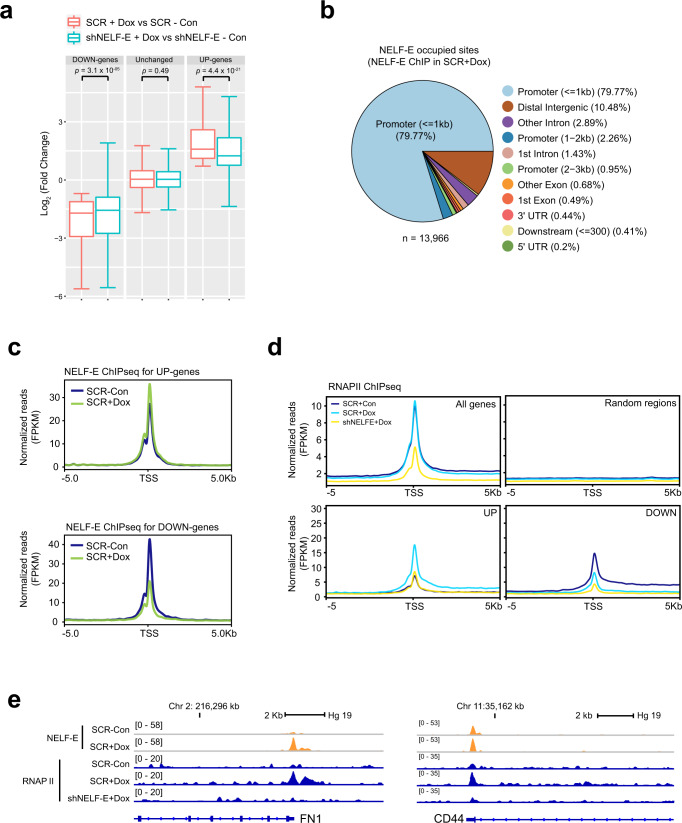
Loss of NELF-E attenuates gene activation and RNAPII binding. **a** Log_2_ fold change of UP-genes (*n* = 1750), DOWN-genes (*n* = 2223), and unchanged genes (*n* = 1581) in the two different conditions. Gene categorization was based on the condition of SCR+Dox *vs* SCR-Con with the cutoffs of adj. *p*-value ≤ 0.05 and |fold change| ≥ 1.5. Center lines show median values, box limits represent the upper and lower quartiles, and whiskers show 1.5× the interquartile range. Two-tailed Student’s *t*-tests were used for all comparisons. *p*-values were not adjusted for multiple tests, and *t*-statistics are provided in source data. **b** Genomic distribution of NELF-E binding sites in Dox-treated MCF7ras+SS cells. **c** Metagene plots of NELF-E ChIP-seq signals in SCR-Con and SCR+Dox cells for UP-genes (top panel) and DOWN-genes (bottom panel). **d** Metagene plots of RNAPII ChIP-seq signals in SCR-Con, SCR+Dox, and shNELF-E+Dox cells for UP-genes and DOWN-genes. **e** Genome browser tracks showing NELF-E and RNAPII occupancies at the promoters of mesenchymal gene *FN1* (left) and stemness-related gene *CD44* (right). Source data are provided as a Source Data file.

From our qPCR analysis, we noticed that the expressions of select mesenchymal and stemness-related genes were significantly attenuated in NELF-E KD Dox-induced MCF7ras+SS cells. However, epithelial genes, especially the well-known marker *CDH1*, remained largely repressed (Supplementary Fig. 4e). To assess this in an unbiased manner, we classified the genes in our RNA-seq data into three groups, based on gene activity changes in SCR+Dox cells compared to SCR-Con cells: 1750 genes that were upregulated (adj. *p*-value < 0.05; fold change ≥ 1.5; referred to as ‘UP-genes’), 2223 genes that were downregulated (adj. *p*-value < 0.05; fold change ≤ −1.5; referred to as ‘DOWN-genes’) and genes whose expression did not change significantly (referred to as ‘unchanged’) (Fig. 3b). Consistent with our earlier findings, loss of NELF-E elicited a greater effect on the UP-genes (*p* = 4.4 × 10^−^^21^), which included the mesenchymal and CSC markers, and a marginal impact on DOWN-genes (*p* = 3.1 × 10^−^^05^) (Fig. 4a). No global effect was observed for unchanged genes (Fig. 4a). This result highlighted a biased effect of NELF-E KD toward the EMT transcriptional response, predominantly affecting SLUG/SOX9-activated target genes.

To determine how NELF promotes the expression of EMT and cancer stemness genes, we performed NELF-E ChIP-seq in control and Dox-induced MCF7ras+SS cells. A total of 14,206 NELF-E peaks were identified in DMSO-treated control cells (SCR-Con) and 13,966 peaks in Dox-treated cells (SCR+Dox) (Fig. 4b and Supplementary Fig. 5a). In general, NELF-E showed similar genomic distribution in both SCR-Con and SCR+Dox cells, largely occupying the promoter-proximal regions (~80% of its total occupied sites) as expected (Fig. 4b and Supplementary Fig. 5a). Notably, the SCR+Dox cells showed a subset of de novo NELF-E binding sites (*n* = 1909) (Supplementary Fig. 5b) that are statistically different between SCR+Dox and SCR-Con cells (Supplementary Fig. 5c). Next, we performed functional enrichment of pathway analysis and found that the de novo NELF-E peaks are also enriched for the EMT pathway (HALLMARK_EMT, adj. *p*-value = 0.0136), represented by the known EMT markers *TIMP3*, *CALD1*, *FAS*, and *IL6* (Supplementary Fig. 5d).

Next, we integrated NELF-E ChIP-seq with RNA-seq data. Overall, the UP-genes showed increased NELF-E binding, whereas DOWN-genes exhibited loss of NELF-E binding at the promoter-proximal regions, indicating that NELF-E occupancy correlated with gene expression level (Fig. 4c). RNAPII occupancy also mirrored that of NELF-E binding (Fig. 4d), as exemplified by cancer stem cell marker *CD44* and mesenchymal gene *FN1*, both of which are upregulated upon Dox induction in control cells but curtailed in NELF-E KD cells (Fig. 4e). These results highlight a positive role of NELF-E-RNAPII partnership in gene activation, and concur with an increasing number of studies showing that the loss of NELF has a modest effect on RNAPII pausing in vivo, but profoundly affects RNAPII occupancy on target genes^23–32^. Taken together, our data emphasize a requirement of NELF-E in promoting the upregulation of genes during EMT induction.

### NELF-E interacts with SLUG and modulates its target occupancy on select loci

To gain insight into the mechanism by which NELF-E promotes EMT, we adopted a proteomics approach to identify interactors of NELF-E. Specifically, we utilized quantitative multiplexed Rapid Immunoprecipitation Mass spectrometry of Endogenous proteins (qPLEX-RIME), a recently developed method to investigate the composition of chromatin-associated complexes in a quantitative manner^44^. We applied NELF-E qPLEX-RIME on both untreated and Dox-treated MCF7ras+SS cells with respective IgG controls (Supplementary Fig. 6a). Analysis of the untreated MCF7ras+SS cells revealed that the NELF-E interactome comprises a total of 89 proteins that showed significant enrichment over IgG controls (log_2_ [fold change] ≥ 0.5; adj. *p*-value < 0.05) (Supplementary Fig. 6b, c and Supplementary Data 3).

NELF-E protein was robustly identified with 26 unique peptides and 65% protein sequence coverage (Supplementary Fig. 6d). The remaining three subunits—NELF-A, NELF-B, and NELF-C/D—were also identified with a high number of unique peptides, along with other known partners of NELF, including RNAPII subunits and NCBP1/2^45^ (Supplementary Fig. 6d). Importantly, many of the identified NELF-E interactors play a role in NELF-E related functions (Supplementary Fig. 6e; see also discussion), further increasing our confidence in the acquired NELF-E interactome.

**Fig. 5:**
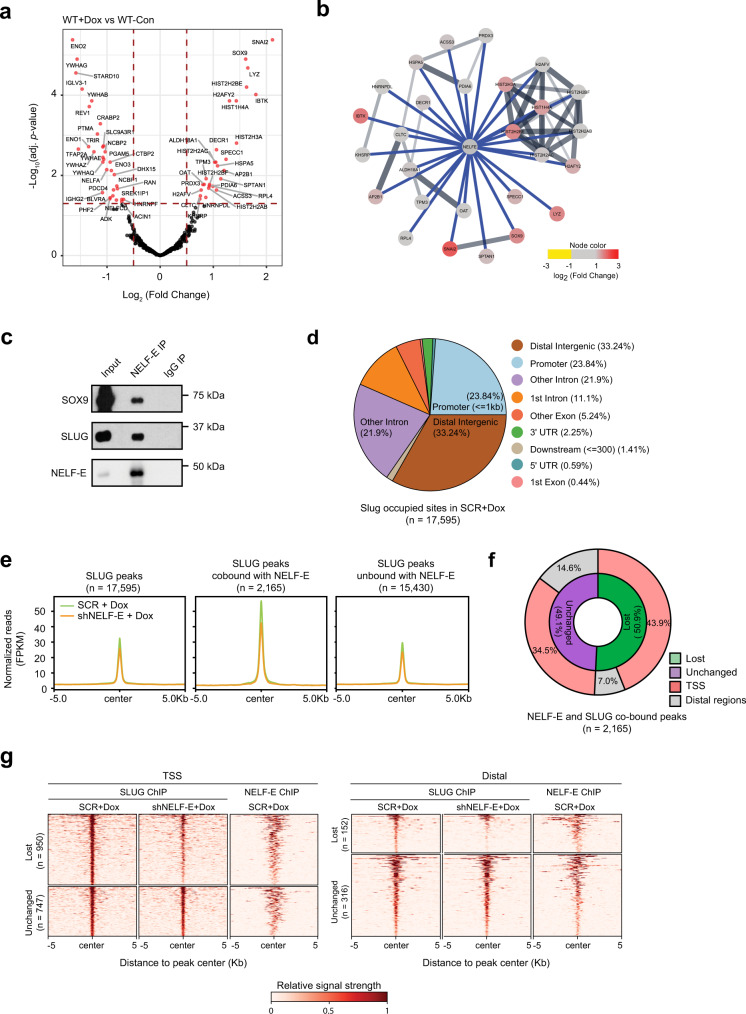
NELF-E interacts with and alters the genomic occupancy of SLUG. **a** Volcano plot of NELF-E qPLEX-RIME analysis of WT+Dox vs WT-Con. Proteins that satisfy the significance threshold of |log_2_(fold change)| ≥0.5 and adj. *p*-value <0.05 are labeled with their gene names and colored red. The *p*-value was adjusted by Benjamini–Hochberg multiple hypothesis correction. **b** Interaction network plot of proteins enriched in Dox induction by qPLEX-RIME, superimposed on the STRING interaction network. Interactions detected by qPLEX-RIME are colored in blue, while interactions from the STRING database are colored in gray. Proteins/nodes are colored based on log_2_(fold change) value. **c** Western blot analysis of SOX9, SLUG, and NELF-E following NELF-E immunoprecipitation (IP), performed on nuclear extracts from Dox-treated MCF7ras+SS cells. Blots are representative of three independent experiments. **d** Genomic distribution of SLUG binding sites in MCF7ras+SS cells transduced with scrambled shRNA and treated with Dox (SCR+Dox). **e** Metaplots of SLUG binding across the three different categories in SCR+Dox and shNELF-E+Dox MCF7ras+SS cells. **f**, **g** Pie chart and heatmap depiction of SLUG and NELF-E co-bound peaks at TSS and distal regions. Source data are provided as a Source Data file.

Next, we sought to delineate changes in the NELF-E interactome upon EMT induction. We analyzed NELF-E qPLEX-RIME on the Dox-treated cells and compared the results to the parental MCF7ras+SS cells. Expression of NELF-E remained unchanged during EMT induction (Fig. 3c), providing a basis to compare protein quantities of identified interactors upon EMT induction. In total, we obtained 27 NELF-E interactors that showed significant enrichment in the EMT state (log_2_ [fold change] ≥ 0.5; adj. *p*-value < 0.05) (Fig. 5a, b, Supplementary Fig. 6f), and 32 NELF-E interactors that showed decreased enrichment in the EMT state (log_2_ [fold change] ≤ −0.5; adj. *p*-value < 0.05) (Supplementary Fig. 6f, g), suggesting a rewired NELF-E interactome during EMT (Supplementary Data 3). We validated some of these interactors in co-immunoprecipitation experiments, and in particular, SLUG and SOX9, which emerged as the top differentially enriched candidate proteins (Supplementary Fig. 7a, Fig. 5c). Importantly, we also recapitulated the interaction between SLUG/SOX9 and NELF-E in another breast cancer cell line, BT-549, that expresses endogenous levels of SOX9 and SLUG, highlighting the generality of our findings (Supplementary Fig. 7b). To our knowledge, SLUG and SOX9 have not been reported as NELF-E interactors, and this finding may thus offer a mechanistic account for how NELF exerts an oncogenic effect through modulating the activities of key EMT TFs. Together, our results constitute the first broad characterization of the NELF-E interactome on chromatin and as a function of EMT, providing insights into the disease mechanisms.

SLUG was previously shown to play a dominant role during EMT induction in the MCF7ras+SS cell line model^40^. Guided by our qPLEX-RIME results, we next examined the genomic occupancy of SLUG in Dox-induced MCF7ras+SS cells, and as a function of NELF-E KD. Upon Dox induction in the parental cells, we detected a total of 17,595 SLUG-bound genomic sites, the majority of which corresponds to regulatory elements such as distal intergenic regions and promoters (Fig. 5d).

To further interrogate the mechanistic relationship between NELF-E and SLUG, we compared the genomic binding profiles of both NELF-E and SLUG in SCR+Dox cells and identified a subset of NELF-E-SLUG overlapping peaks (*n* = 2165). Interestingly, SLUG occupancy is highest in NELF-E bound regions, which is reduced following NELF-E depletion (Fig. 5e), as exemplified by the *PHTF1 and MANBA* genomic loci (Supplementary Fig. 7c**)**. Importantly, this reduced occupancy is not due to changes in SLUG protein expression upon NELF-E KD (Fig. 3c). We additionally determined through differential peaks analysis, followed by statistical testing, that these co-bound peaks are more likely to lose SLUG binding upon NELF-E depletion, compared to the cohort of SLUG peaks that are not co-bound by NELF-E (chi-square test; observed to expected ratio: 3.4, *p*-value = 1.77 × 10^−^^261^). Moreover, a majority (78.4%) of the NELF-E-SLUG co-bound peaks are located at promoters, and NELF-E depletion resulted in a greater loss of SLUG co-bound peaks at promoters, compared to distal regions (43.9% vs 7.0%) (Fig. 5f, g). Importantly, SLUG binding at NELF-E de novo regions is also reduced upon NELF-E KD (Supplementary Fig. 7d). Taken together, these data highlight an important role of NELF-E in regulating SLUG genomic occupancy, in part through its interaction with SLUG on chromatin. Notably, we also validated in another breast cancer cell line, BT-549, that depletion of NELF-E led to the diminution of a large number of SLUG binding sites (Supplementary Fig. 7e**)**. Next, we plotted NELF-E and SLUG signals in the ‘SLUG-unchanged’ and ‘SLUG-lost’ categories (SCR vs shNELF-E BT-549 cells). Interestingly, we found that although NELF-E binding strength is comparable across these two categories, SLUG regions with higher intensity are more sensitive to NELF-E loss (Supplementary Fig. 7f), supporting our mechanistic model.

### KAT2B is a key functional target of NELF-E-SLUG during EMT

**Fig. 6:**
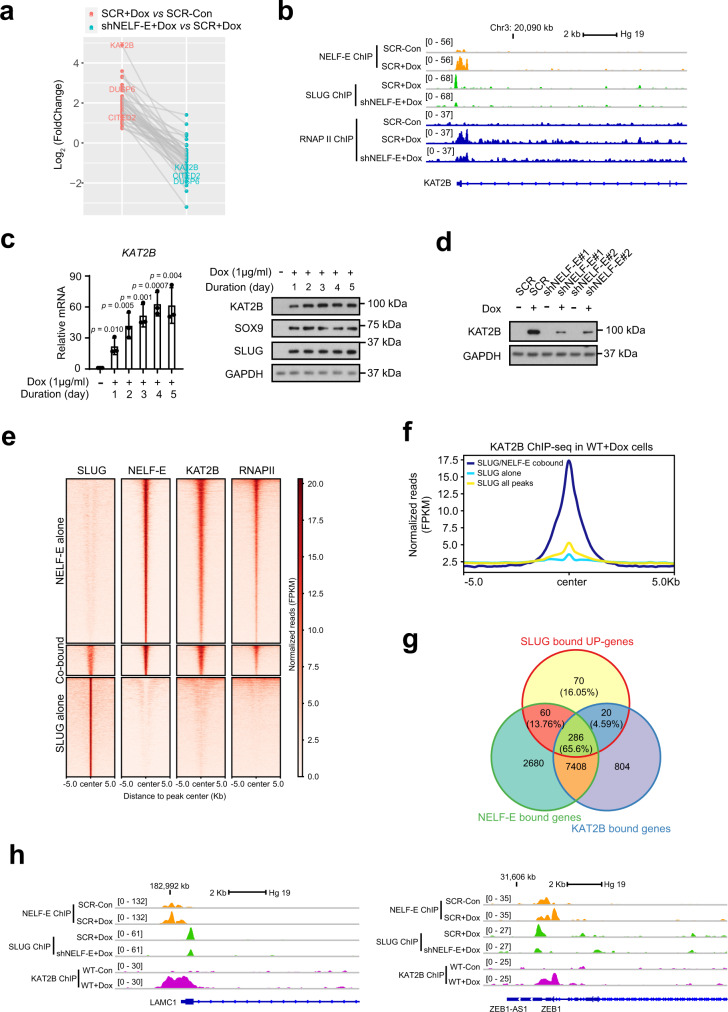
KAT2B is a key target of NELF-E and SLUG in response to EMT. **a** Dot plot showing KAT2B as one of the top genes (*n* = 41) whose SLUG-mediated activation was most significantly attenuated by NELF-E KD. Expressions of corresponding genes in the two comparisons are connected by gray lines. **b** Genome browser tracks showing the occupancies of NELF-E, SLUG, and RNAPII on the *KAT2B* locus. **c** RT-qPCR analysis (left) and western blot analysis (right) of KAT2B in MCF7ras+SS cells treated with Dox for different time points (*n* = 3). **d** Western blot analysis of KAT2B in MCF7ras+SS cells transduced with scrambled shRNA or two independent NELF-E shRNAs and treated with or without Dox. GAPDH was used as the loading control. **e** Heatmap depiction of SLUG/NELF-E/KAT2B/RNAPII signals at NELF-E-alone, SLUG-alone and NELF-E/SLUG co-bound regions. **f** Metaplot of KAT2B ChIP-Seq signals over the different cohorts of SLUG-bound regions. **g** Venn diagram showing the overlap between SLUG-bound UP-genes and NELF-E/KAT2B targets. The percentage overlap of SLUG-bound UP-genes compared to other categories is also indicated. **h** Genome browser tracks showing the occupancies of NELF-E, SLUG, and KAT2B on *LAMC1* and *ZEB1* loci. Blots are representative of three independent experiments. *p*-values are determined by a two-tailed Student’s *t*-test. Mean ± SD is represented by bar graphs. Source data are provided as a Source Data file.

To elucidate the direct targets of both NELF-E and SLUG that contribute to the induction of the mesenchymal/stemness gene expression program, we annotated the NELF-E-SLUG co-bound peaks to their nearest genes within a 10 kb linear distance. To identify genes that are most profoundly affected by the loss of NELF-E upon SLUG/SOX9 overexpression, we defined a NELF-E KD impact score (IS) by subtracting the normalized log_2_(fold change) of SCR+Dox vs SCR-CON from that of shNELF-E+Dox vs shNELF-E-Con. A negative IS indicates that NELF-E KD either attenuates gene activation or enhances gene inhibition by SLUG/SOX9 overexpression, while a positive IS indicates that NELF-E KD either enhances gene activation or attenuates gene inhibition in response to SLUG/SOX9 overexpression. We then filtered and kept genes whose activation by SLUG overexpression is impaired upon NELF-E KD by imposing the criterion that these genes are significantly upregulated in Dox-induced MCF7+SS cells (fold change ≥ 1.5 and adj. *p*-value < 0.05) and their NELF-E KD IS is no larger than −log_2_(1.5) (corresponding to a 1.5-fold reduction in activation). By overlapping these two gene sets, we arrived at 41 genes that are direct targets of NELF-E and SLUG, and whose expressions were significantly attenuated upon NELF-E KD (Fig. 6a and Supplementary Data 4). From this analysis, we identified genes such as CITED2 and DUSP6 that are previously implicated in breast cancer metastasis^46,47^. Notwithstanding that SLUG is a well-known transcriptional repressor^48,49^, our finding highlights that it can also exert an activation function on a subset of target genes in association with NELF-E.

Among the 41 NELF-E-SLUG-activated targets, we found *KAT2B* to be the most highly upregulated gene (Fig. 6a). KAT2B is a lysine acetyltransferase that catalyzes lysine acetylation on histones and non-histone proteins^50^. It is implicated in a variety of cellular functions, including tumorigenesis^51^. Notably, the induction of EMT is accompanied by extensive chromatin changes, and in particular, the acquisition of euchromatic features^52–54^. Moreover, in an independent single-cell RNA-seq dataset^55^, we observed that *KAT2B* mRNA positively correlates with signature mesenchymal genes upon EMT induction (Supplementary Fig. 8a). We therefore decided to focus on KAT2B and determine how it contributes to breast cancer progression.

We first confirmed that NELF-E and SLUG are recruited to the *KAT2B* promoter upon Dox induction (Fig. 6b) and is accompanied by an increase in KAT2B expression (Fig. 6c and Supplementary Fig. 8b). Importantly, KD of NELF-E led to reduced KAT2B expression, as well as decreased SLUG and RNAPII occupancies at the *KAT2B* promoter, indicating that NELF-E contributes to the stabilization of SLUG and RNAPII binding at the *KAT2B* promoter (Fig. 6b, d and Supplementary Fig. 8b). Whereas the upregulation of *KAT2B* was impaired in the absence of NELF-E, the expression of its paralogue *KAT2A* was not significantly affected (Supplementary Fig 8c). These results established KAT2B as a direct transcriptional target of NELF-E and SLUG.

### KAT2B synergizes with NELF-E-SLUG to promote EMT and stemness

Having demonstrated a dependency on NELF-E and SLUG for KAT2B expression, we proceeded to assess the mechanistic interplay between KAT2B and NELF-E-SLUG during EMT. We characterized the genomic localization of KAT2B by performing KAT2B ChIP-seq in control and Dox-induced MCF7ras+SS cells, which revealed a predominant occupancy of KAT2B at promoters upon Dox induction (Supplementary Fig. 8d). Pathway analysis indicated that genes targeted by KAT2B were involved in processes related to EMT, such as MYC, E2F, and TGFβ signaling, thereby supporting a pro-tumorigenic role of KAT2B (Supplementary Fig. 8e). Interestingly, we also found that the overall occupancy of KAT2B strongly correlated with that of NELF-E genome-wide (Fig. 6e), and KAT2B occupancy was significantly enriched across SLUG binding sites only if co-bound by NELF-E (Fig. 6f). These observations highlight a potential synergistic relationship between NELF-E and KAT2B in gene activation, including on select SLUG-target genes.

To further explore an activation function of SLUG, we stratified SLUG-target genes based on their transcriptional status. We obtained a total of 1207 SLUG-repressed genes and 436 SLUG-activated genes (Supplementary Fig. 8f). Focusing on the latter, we confirmed that SLUG-activated genes are enriched for the EMT pathway (Supplementary Fig. 8g), and about 65.6% (*n* = 286) of the genes are co-occupied by NELF-E and KAT2B (Fig. 6g). Notable examples include *ZEB1*, a major inducer of EMT, as well as *LAMC1*, an extracellular matrix protein associated with tumor invasion and metastasis (Fig. 6h). Further to their genomic co-occupancy, we also detected a biochemical interaction between KAT2B and SLUG (Supplementary Fig. 8h). Taken together, our data reveal mechanistic insights into how KAT2B drives breast cancer metastasis and contributes to SLUG-mediated gene activation in association with NELF.

**Fig. 7:**
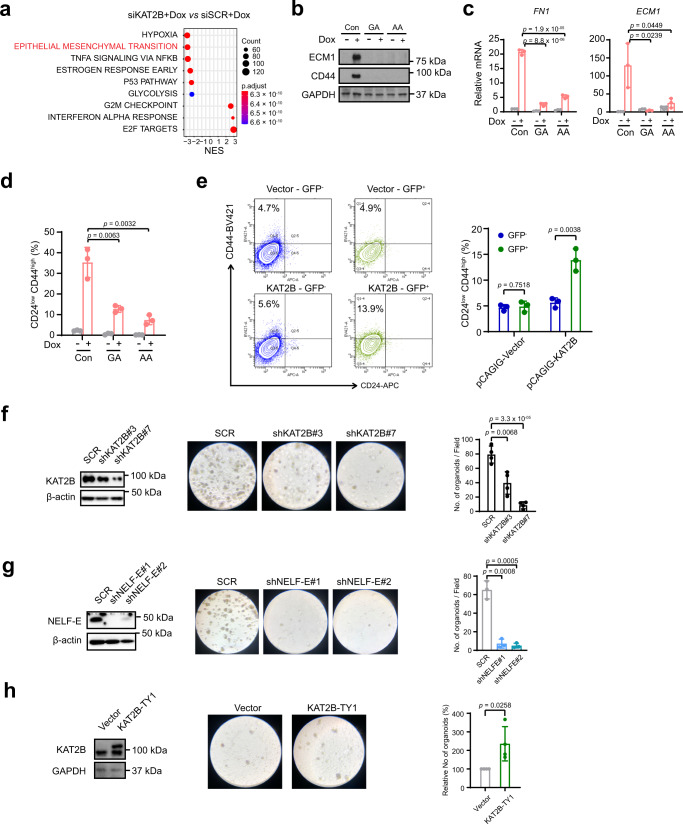
KAT2B activates EMT progression. **a** GSEA plot showing significantly enriched pathways in siKAT2B+Dox vs siSCR+Dox MCF7ras+SS cells. *p*-value was calculated from gene set enrichment analysis (see details in “Methods”). **b** Western blot analysis of ECM1 and CD44 in MCF7ras+SS cells treated with or without GA and AA. GAPDH was used as the loading control. Images and blots are representative of three independent experiments. **c** qRT-PCR analysis of *FN1* and *ECM1* in GA- and AA-treated Dox-induced MCF7ras+SS cells (*n* = 3). **d** Flow cytometry analysis and quantification of the CD24^low^/CD44^high^ population in vehicle control, GA-, and AA-treated MCF7ras+SS cells, as a function of Dox induction. **e** Flow cytometry analysis and quantification of the CD24^low^/CD44^high^ population following KAT2B overexpression in MCF7ras+SS cells. Vector control (GFP only; pCAGIG) and KAT2B-overexpression (KAT2B+GFP; pCAGIG-KAT2B) plasmids were transfected into MCF7ras+SS cells, respectively. The GFP^−^ and GFP^+^ populations were isolated and analyzed for the CD24^low^/CD44^high^ population (*n* = 3). **f**, **g** Patient-derived breast cancer organoids were transduced with scrambled shRNA or two independent KAT2B or NELF-E shRNAs, followed by western blot and sphere-formation assay (*n* = 3). β-actin was used as the loading control. **h** Patient-derived breast cancer organoids were transduced with vector control or KAT2B overexpressing plasmid, followed by western blot and sphere-formation assay (*n* = 4). GAPDH was used as the loading control. Blots and images are representative of at least three independent experiments. *p*-values in (**c**–**h**) are determined by a two-tailed Student’s *t*-test. Mean ± SD is represented by bar graphs. Source data are provided as a Source Data file.

To investigate the functional consequences of KAT2B ablation on EMT and cancer stemness, we depleted KAT2B in the Dox-induced MCF7ras+SS cells. RNA-seq followed by pathway analysis of the KAT2B KD cells revealed that EMT and related pathways (e.g., NF-κB) are most significantly impacted (Fig. 7a). Western blot analysis further confirmed the loss of the stemness-associated marker, CD44 (Supplementary Fig. 9a). Importantly, we also validated that loss of KAT2B led to impaired breast cancer stemness in a different metastatic breast cancer cell line, BT-549 (Supplementary Fig. 9b).

Further to the genetic loss-of-function experiments, we also sought to disrupt KAT2B activity using the pharmacological inhibitors, Garcinol (GA) and anacardic acid (AA)^56,57^. We first determined the optimal working concentrations of GA and AA that did not induce toxicity in MCF7ras+SS cells (Supplementary Fig. 9c). Importantly, Dox-induced MCF7ras+SS cells treated with either GA or AA showed reduced expression of mesenchymal markers such as CD44, FN1, and ECM1, as well as the CSC cell surface marker CD24^low^ CD44^high^ (Fig. 7b–d), phenocopying our KAT2B KD results. We subsequently performed RNA-seq on GA-treated cells and observed that the EMT pathway was downregulated (Supplementary Fig. 9d) and that a significant number of downregulated genes in KAT2B-inhibited cells was similarly affected upon NELF-E KD, consistent with the extensive genomic co-occupancy of both factors (Supplementary Fig. 9e). This is further supported by a correlation analysis of the whole transcriptomes comparing GA-treated and NELF-E KD cells, which showed a significant positive correlation (rho = 0.15, *p* < 2.2 × 10^−^^16^). A stronger positive correlation was observed when using the HALLMARK_EMT gene signatures (rho = 0.33, *p* = 7.1 × 10^−^^7^) (Supplementary Fig. 9f). Strikingly, NELF-E genomic occupancy was reduced upon GA treatment, including at EMT-related genes, consistent with their transcriptional downregulation (Supplementary Fig. 9g, h). Taken together, our findings uncovered a cooperative function between NELF-E and KAT2B in regulating EMT genes.

To definitively ascribe the role of KAT2B in promoting an EMT/CSC-like state, we performed gain-of-function experiments, in addition to the aforementioned loss-of-function studies. Consistent with our findings thus far, overexpression of KAT2B in control MCF7ras+SS cells led to an increase in the CD24^low^/CD44^high^ CSC population (Fig. 7e and Supplementary Fig. 9i). Hence, our data strongly demonstrate that upregulation of KAT2B contributes to a malignant CSC-like state.

To substantiate the role of KAT2B as a key effector of breast tumorigenesis, we evaluated the expression of *KAT2B* and mesenchymal signature markers in patient tumors using various BRCA datasets and observed a consistent positive correlation with ZEB1/2 (Supplementary Fig. 9j). Furthermore, higher protein expression of KAT2B also correlated with a poorer survival outcome of breast cancer patients (Supplementary Fig. 9k).

Last but not least, beyond cancer cell lines, we also knocked down KAT2B in a TNBC patient-derived organoid cell line and observed impaired mammosphere-forming ability, which is recapitulated by NELF-E KD (Fig. 7f, g). Conversely, overexpression of KAT2B promoted mammosphere formation (Fig. 7h). In sum, our work illustrates the crucial role of the NELF-KAT2B epigenetic axis in breast cancer carcinogenesis, particularly in the context of EMT and cancer stemness.

## Discussion

Chromatin-associated proteins are emerging as new therapeutic targets for a variety of diseases, especially cancer. Here, we demonstrated that loss of the NELF complex abolished tumorigenic properties in breast cancer cells irrespective of their histological subtypes, revealing a crucial role of NELF in breast cancer carcinogenesis. Specifically, we showed that NELF activates the EMT and mammary stem cell transcriptional programs in breast cancer, and defined an epigenetic mechanism by which NELF co-opts the EMT TF, SLUG, and histone acetyltransferase, KAT2B, to coordinate gene expression reprogramming during EMT.

Much of our current understanding of NELF’s function is derived from in vitro biochemical studies. However, in vivo genetic studies have revealed additional functions of NELF independent of transcriptional pausing, as well as a more complex role in gene regulation that is likely influenced by the chromatin context in which NELF functions. To enable an in-depth exploration of NELF’s function on chromatin, we applied the qPLEX-RIME method to define the NELF-E ‘chromatome’ during EMT progression, which revealed a comprehensive network of interactions. Importantly, not only did we recover members of the paused transcription complex (RNAPII–DSIF–NELF) as expected, but we also identified numerous novel partners that may reveal new insights into NELF’s function in vivo. Many of the identified NELF-E interactors likely play roles in NELF-E-related functions. For instance, the 89 identified NELF-E interactors include proteins involved in RNA transcription and cofactors (TAF15, DDX21, SUB1), mRNA splicing (DHX15, SRSF7, SRSF8, PUF60), mRNA methylation (CMTR1), and telomerase RNA processing (TRIR), pointing to a wider function of NELF in transcription regulation (Supplementary Data 3). Proteins involved in DNA repair (PCNA, RUVBL1) were also identified as NELF-E interactors, consistent with recent reports identifying NELF as a new player in DNA damage response^58,59^. In addition, chromatin binding proteins (CBX3, HMGB1) and proteins involved in post-translational modifications on histones (PRMT1, KMT5A) were also uncovered as NELF-E interactors, and these are likely linked to gene regulatory functions of the NELF complex. Notably, other proteins with no obvious links or prior connections to NELF function were also identified. For example, all three enolase isoenzymes, ENO1–3, which are primarily known for their roles in glucose metabolism, were pulled down, hinting at a possible moonlighting function for these metabolic enzymes in the nucleus in association with NELF. Interestingly, ENO1 was previously reported to bind to chromatin and may function as a transcriptional regulator^60^. While the functional importance of these interactions remains to be investigated, our work provides an important resource and framework to study NELF’s function and its mechanisms of action in cancer.

Another key observation that emerged from our study is that there is a significant rewiring of the NELF-E interactome following EMT induction. SLUG and SOX9 were identified as enriched upon EMT induction in the NELF-E interactome, together with other factors such as IBTK and histones. In our EMT cell line model, the NELF-E level remains unchanged upon EMT induction, suggesting that NELF is hijacked by oncogenic TFs to promote the transcriptional plasticity necessary to drive cellular reprogramming for the acquisition of metastatic properties. Notably, EMT is a highly dynamic process that encompasses a wide spectrum of intermediate states. This plasticity endows cancer cells with a greater potential for cancer progression, dissemination, and adaptation to selective pressures^61,62^. SNAIL, SLUG, TWIST1, ZEB1, and ZEB2 are widely considered core EMT TFs. Interestingly, these factors are known to exhibit different spatiotemporal expression patterns in tumors and act in a non-redundant fashion to activate the EMT program^63^. Depending on the tumor types, context-specific nuances exist and different sets of EMT TFs may be utilized. It is important to highlight that in our cell line models, we primarily focused on NELF-E’s partnership with SLUG. It is possible that NELF-E may also partner with SOX9 for robust activation of the EMT program. We further hypothesize that NELF may be aberrantly co-opted by different EMT TFs to drive the reprogramming of gene expression during distinct stages of EMT, and if so, may represent an important therapeutic vulnerability in metastatic cancers in general. Indeed, further to our study in breast cancer, NELF has also been shown to promote metastasis in other cancers such as pancreatic cancer^64^, liver cancer^65^, and gastric cancer^66^.

Although SLUG is well known as a transcriptional repressor, our study also draws attention to its role in gene activation. Here, by integrating both transcriptome and ChIP-seq datasets, we discovered numerous NELF-E-SLUG-activated targets, many of which are directly implicated in EMT, such as *ZEB1* and *KAT2B*. The observed genomic co-occupancy of KAT2B on NELF-E-SLUG-bound chromatin regions, along with our biochemical interaction studies, further suggests a model in which these factors cooperate extensively to establish a transcriptionally competent chromatin state for metastatic gene expression. This is corroborated by a previous study that reported SLUG overexpression being linked to increased histone acetylation on the promoters of EMT genes^67^. Furthermore, KAT2B can form an activating complex with ZEB1 to stimulate the transcription of EMT targets via histone acetylations^68^. Mechanistically, we envisage that as a TF, SLUG is recruited to its target promoters primarily through a DNA sequence-directed manner, and NELF may help to stabilize SLUG binding on chromatin at co-bound sites, likely by recruiting the transcription machinery, as suggested by our qPLEX-RIME. NELF further partners with KAT2B to facilitate SLUG-mediated activation of EMT genes.

Although our study largely focused on the NELF-E-SLUG co-bound genes, we wish to highlight that loss of NELF-E can have a broader impact on SLUG binding genome-wide. SLUG is known to cooperate with various cofactors for its binding and transcriptional activity^69,70^. From our transcriptomic analysis, we noticed that several cofactors, such as *JUNB* and *TCF3*, are downregulated upon NELF-E KD (Supplementary Data 2), which may account for the observed reduction in SLUG occupancy outside of NELF-E co-bound sites. Thus, our findings draw attention to the different regulatory mechanisms employed by NELF to impact SLUG targeting, and possibly that of other EMT TFs as well. In future studies, we aim to elucidate the mechanistic basis by which NELF modulates changes in the genomic occupancy of different EMT TFs to control their respective EMT programs.

In conclusion, our results highlight how NELF-E-SLUG-KAT2B regulatory axis can be exploited by breast cancer cells to drive phenotypic plasticity, culminating in cancer progression and metastasis. Our findings are consistent with mounting evidence demonstrating that cancer cells utilize mechanisms to support an enhanced transcriptional activity that is required to sustain their oncogenic programs, rendering them sensitive to transcriptional-based inhibition. While our study specifically examined the transcriptional effects of NELF in breast cancer carcinogenesis, a previous study reported that NELF-E may also exert a post-transcriptional function by modulating the mRNA stability of oncogenic transcripts in liver cancer^71^. Notwithstanding the different mechanisms by which NELF may promote tumorigenesis, these findings clearly underscore the critical pro-oncogenic abilities of NELF in different cancers.

Breast cancer is one of the top cancers among women worldwide, and metastasis is mainly responsible for treatment failure, accounting for the majority of breast cancer-associated mortality. Unfortunately, the efficacy of currently available systemic therapies in advanced-stage breast cancer and relapsed disease has remained limited. From a therapeutic perspective, it may be possible to target the NELF-E-SLUG-KAT2B dependency in breast cancer. Importantly, a previous genetic KO study showed that adult mice lacking a functional NELF complex are viable, albeit with impaired response to cardiac stress, at later stages of adulthood^72^. This indicates that NELF is largely dispensable for basal gene expression in normal tissues, in contrast to the prevailing view that transcriptional regulators are ‘housekeeping’ in function. Therefore, there is likely a sufficient therapeutic window to target NELF in cancer. Similarly, *Kat2b* KO mice are viable with no obvious detrimental phenotype^73^, enabling it to serve as a potential clinically actionable therapeutic target. The apparent non-essentiality of KAT2B and NELF for normal development, together with their acquired dependency in aggressive breast cancer, highlight the exciting prospect of developing therapeutic candidates to target NELF-E-KAT2B in cancer.

## Methods

### Ethical statement

Our research complies with all relevant ethical regulations. All animal experiments were approved by the Institutional Animal Care and Use Committee (IACUC; 181412 and 201572), Agency for Science, Technology and Research (A*STAR), Singapore. Generation of patient derived breast cancer organoid was done in compliance with all relevant ethical regulations, with informed consent from the patient, and approved by the National Healthcare Group DSRB (reference number: 2015/00357).

### Cell lines and cell culture

MCF7 (ATCC, HTB-22) cells were obtained from ATCC and maintained in Dulbecco’s Modified Eagle Medium (DMEM) supplemented with 10% (v/v) fetal bovine serum (FBS) and 1% (v/v) penicillin/streptomycin. BT-549 (ATCC, HTB-122) and T-47D (ATCC, HTB-133) cells were obtained from ATCC and grown in RPMI-1640 medium supplemented with 10% (v/v) FBS and 1% (v/v) penicillin/streptomycin. MCF7, BT-549, and T-47D were further verified by short tandem repeat (STR) transcriptomic profiling. SUM159PT (Bioivt, HUMANSUM003006; referred to as SUM159) cells were obtained from Bioivt and maintained in Nutrient Mix F12 (HAM) medium supplemented with 10% (v/v) heat-inactivated FBS and 1% (v/v) penicillin/streptomycin. HMEC cell line (Lonza, CC-2551) was a gift from Su Chin Tham (IMCB) and grown in MEBM^TM^ Basal Medium (Lonza, CC-3151) supplemented with MEGM^TM^ SingleQuots^TM^ Supplements (Lonza, CC-4136). MCF7ras+SS cell line was a gift from Dr. Wai Leong Tam (GIS) and Dr. Elina Pathak (GIS) and grown in DMEM supplemented with 10% (v/v) fetal bovine serum (FBS) and 1% (v/v) penicillin/streptomycin, which was validated in a previous publication^40,41^. BT-474 (ATCC, HTB-30) and SK-BR-3 (ATCC, HTB-30) cells were gifts from Dr. Boon Tin Chua and grown in RPMI-1640 medium and McCoy’s 5A medium, respectively supplemented with 10% (v/v) FBS and 1% (v/v) penicillin/streptomycin. Cells were tested routinely for mycoplasma and were free of contamination at the point of our experiments.

### RNA isolation and real-time PCR

Total RNA was isolated using TRIzoland Direct-zol RNA miniPrep Kit (ZYMO research, R2052) following the manufacturer’s protocol. cDNA was generated using the SensiFAST cDNA Synthesis Kit (BIOLINE, BIO-65054). Quantitative real-time PCR with Powerup SYBR Green PCR Master Mix (Life Technologies, A25742) was performed on the QuantiStudio5 Real-time PCR System (Applied Biosystems). Primer sequences are shown in Supplementary Data 5.

### Generation of CRISPR/Cas9 knockout (KO) cells

The all-in-one expression plasmid system pSpCas9(BB)–2A-GFP (PX458) containing Green Fluorescent Protein (GFP) reporter, Cas9, and sgRNA was obtained from Addgene (RRID: Addgene_48138). The target DNA sequences for NELF-A and NELF-E sgRNAs are shown in Supplementary Data 5. MCF7 and SUM159 cells were transfected with PX458 containing sgRNA using Lipofectamine 3000 (Invitrogen). At 48 h after transfection, GFP-negative (Control cells) and GFP-positive cells were sorted and cultured in 96-well plates. Single-cell clones were grown and expanded for western blot analysis. Only the clones that showed depletion of NELF-E protein were selected and expanded for subsequent studies. Positive clones were also verified by Sanger sequencing.

### Small interfering RNA (siRNA) transfection

siRNAs were purchased from Dharmacon and transfected into cancer cells using Lipofectamine RNAiMAX (Thermo Fisher Scientific, 13778150) according to the manufacturer’s instructions. Forward and reverse transfections (40 nM per transfection as final concentration) were carried out, and the effect of the knockdown was analyzed at 72 h after the first transfection. The siRNAs are listed in Supplementary Data 6.

### Preparation of cell lysates and western blots

Cells were washed with phosphate-buffered saline (PBS) and lysed in urea lysis buffer (50 mM TRIS pH 7.9, 8 M Urea, 1% (w/v) CHAPS) containing protease and phosphatase inhibitors (1 mM Aprotinin, 1 mM Pepstatin, 1 mM Leupeptin, 2 mM phenylemethane sulfonyl fluoride (PMSF), 3 mM sodium butyrate and 1X Xpert Phosphatase Inhibitor Cocktail Solution). Protein assay dye reagent concentrate (Bio-Rad; Catalog No. 5000006) was used to measure the lysate protein concentration. Then, 10–40 µg of boiled protein lysates were separated on an 8–12% Bis-Tris PAGE gel before trans-blotting onto a PVDF membrane. The membrane was blocked with 3% (w/v) BSA for 1 h before incubating with respective primary antibodies overnight at 4 °C. The primary antibodies are listed in Supplementary Data 7. Blots were washed and then incubated with respective HRP-conjugated secondary antibodies for 1 h at room temperature. Blots were developed by the ECL-based chemiluminescence method (Bio-Rad; Catalog No. 170–5061) and imaged using the film developer, Chemidoc touch imaging system (Bio-Rad) or Invitrogen™ iBright™ Imaging Systems.

### Selection of NELF-E knockdown cells

To generate lentiviruses, two independent NELF-E shRNAs were cloned into the pLKO.1-puro vector (Dharmacon; RHS3979-201790329 and RHS3979-201792908), and three independent KAT2B shRNAs (Dharmacon; RHS3979-201749903, RHS3979-201749906, and RHS3979-201749907) were cloned into the pLKO.1-blast vector (Supplementary Data 8). These constructs, as well as their scrambled controls, were co-transfected into HEK293T cells along with the third-generation lentivirus helper plasmids (pMDLg/pRRE, pRSV-Rev, and pMD2.G plasmids). Lentiviruses were harvested 48 h after transfection and concentrated prior to transduction. T-47D cells were transduced with two independent lentiviruses together, and stable knockdown cells were selected in a medium containing 2 µg/ml puromycin for at least 5 days. In addition to T-47D, MCF7ras cells were transduced with two independent lentiviruses individually, and stable knockdown cells were selected in a medium containing 5 µg/ml puromycin for at least 3 days. BT-549 cells were transduced with three independent lentiviruses individually, and stable knockdown cells were selected in a medium containing 1 µg/ml blasticidin for at least 3 days.

### Nuclear extraction and co-Immunoprecipitation (co-IP)

Nuclear extracts were prepared and co-IP was performed as described previously with minor modifications^17^. Briefly, MCF7ras+SS cells treated with Dox for 72 h were collected and resuspended in ice-cold TMSD buffer (20 mM HEPES (pH 7.5), 5 mM MgCl_2_, and 250 mM sucrose containing 1 mM dithiothreitol (DTT) and protease inhibitors). Next, cells were resuspended and incubated with ice-cold TMSD buffer containing 0.1% (v/v) Nonidet P-40 (NP-40) to release the nuclei. The nuclei were then immediately lysed with ice-cold low salt lysis buffer (20 mM Tris-Cl (pH 7.9), 420 mM KCl, 1.5 mM MgCl_2_, and 0.2 mM EDTA) with protease inhibitors. Nuclear lysis was carried out at 4 °C with constant rotation, followed by three-cycle sonication in a Bioruptor (Diagenode). Thereafter, the nuclear lysate was spun down at maximum speed, and the supernatant (nuclei lysis) was transferred into a fresh microfuge tube. The insoluble pellet from the initial nuclear lysis was subjected to an additional round of extraction with ice-cold high salt lysis buffer (20 mM Tris-Cl (pH 7.9), 700 mM KCl, 1.5 mM MgCl_2_, and 0.2 mM EDTA) with protease inhibitors. This lysis step was similarly carried out at 4 °C with constant rotation and with three-cycle sonication. The lysate was spun at maximum, and the supernatant (nuclei lysis) was then transferred into a fresh microfuge tube. Both lysates were dialyzed in BC100 (50 mM Tris-Cl (pH 7.9), 2 mM EDTA, 10% (v/v) glycerol, 100 mM KCl, and 0.2 mM PMSF) and combined. Then, 500 μg–1 mg of lysates were used per immunoprecipitation and 10 μg of lysates were used as input. For each immunoprecipitation, 4 μg of NELF-E (Abcam, ab170104) and normal rabbit IgG (Cell Signaling Technologies, 2729) antibodies were used per mg of nuclear extracts respectively. Immunoprecipitation was carried out overnight with constant rotation at 4 °C. Then, 50 μl protein-G Dynabeads (Thermo Fisher Scientific; catalog number 10003D) was added to each immunoprecipitation reaction to capture the antibody–antigen complex. This was followed by five washes in BC200 (50 mM Tris-Cl (pH 7.9), 2 mM EDTA, 10% (v/v) glycerol, 200 mM KCl, and 0.1% (v/v) NP-40). Immunoprecipitations were finally eluted with 2×Laemmli buffer and boiled for 10 min. Boiled samples were then placed on a magnetic stand, and the eluates were transferred into a fresh tube before setting a standard western analysis.

### Flow cytometry analysis

Cancer stem cell-like population was detected using CD44 and CD24 markers. Adherent cells and spheroids were dissociated into single cells and blocked by 2% (w/v) BSA. Cells were labeled with either FITC-conjugated CD24 antibody (BD Biosciences, 560992) or APC-conjugated CD24 (Invitrogen, 17-0247-41), and PE-conjugated CD44 antibody (BD Biosciences, 550989) or BV421-conjugated CD44 (Biolegend, 338809) following the manufacturer’s instructions. All samples were assessed and analyzed by the BD LSR II Flow Cytometer.

### Immunofluorescence staining

MCF7ras+SS cells treated with vehicle or Dox for 72 h were fixed in 4% PFA for 10 min at room temperature and then permeabilized and blocked in blocking buffer (0.1% (v/v) Triton X-100, 1% (w/v) BSA in 1×phosphate-buffered saline (PBS)) for 30 min. Fixed cells were incubated with PCAF/KAT2B antibody (Santa Cruz, sc-13124) overnight at 4 °C. On the following day, cells were washed in PBS three times and then stained with anti-mouse secondary antibody (Thermo Fisher, A32766) at room temperature for 1 h, followed by three washes with PBS. DAPI (STEMCELL, 75004) was used as a nuclear counterstain. Images were taken using a Zeiss LSM700 Inverted Confocal.

### Colony formation assay

For this, 2 × 10^3^ cells dissociated from MCF7 tertiary spheroids were seeded into 6-well plates for 15 days and media were changed every 3 days. Colonies were fixed in cold 70% ethanol and stained with 0.05% crystal violet (Sigma, C0775-25G).

### Wound healing assay

Cells were seeded into 6-well dishes to make a confluent monolayer. The cell monolayer was then scraped in a straight line with P10 pipet tips. Extra wash with the medium was performed to remove debris. The scratched areas were photographed immediately with a phase-contrast microscope at labeled positions. After incubation at 37 °C for 12 h, images were taken for the same areas. The scratched areas were analyzed quantitatively using the Zen Blue software (Zeiss). The distance between opposite edges of the wound was measured using the distance measurement tool in the software. The percentages of wound coverage were calculated according to its original distance at the starting time point.

### Migration assay

For this, 1 × 10^5^ cells in 300 µl of serum-free medium were seeded in the upper chamber of trans-well (CORNING, 353097) with medium containing 5% FBS in the bottom layer. After incubation at 37 °C for 48 h, cells were fixed in ice-cold 70% ethanol and then stained with 0.5% (w/v) crystal violet. Cells on the upper surface of the trans-well were gently wiped off before imaging. Representative fields were selected for imaging, and data was analyzed using Image-J.

### Invasion assay

Trans-well inserts (CORNING, 353097) were coated with 1 mg/ml of diluted Matrigel (CORNING, 356230) at least 30 min prior to this assay. Then, 2 × 10^5^ cells in 300 µl of serum-free medium were seeded in the upper chamber of trans-well with medium containing 5% FBS in the bottom layer. After incubation at 37 °C for 48 h, cells were fixed in ice-cold 70% ethanol and then stained with 0.5% (w/v) crystal violet. Cells on the upper surface of the trans-well were gently wiped off before imaging. Representative fields were selected for imaging, and data was analyzed using Image-J.

### Anchorage-independent growth analysis

For this, 600 µl medium containing 0.6% agar was added into each well of a 24-well plate as the bottom layer. Next, cells in 500 µl medium containing 0.3% agar were layered as the media layer. Then, 500 µl medium was added to each well. Cells were incubated for approximately 1 month to grow into visible colonies, and stained using MTT. Images were taken under a phase-contrast stereomicroscope (Nikon, SMZ645).

### Sphere-formation assay

Spheroids were dissociated in 100 µl of MammoCult™ Human Medium (STEMCELL Technologies) and seeded into 96-well ultra-low attachment plates (Corning, 7007). Cell viability of each spheroid was measured after 10–14 days by using CellTiter-Glo 3D luminescence assay (Promega, G9683).

To measure the number of spheroids, dissociated cells were grown in 6-well ultra-low attachment plates (Corning, 3471). Sphere media was first prepared by serum-free DMEM-F12 (Gibco), supplemented with B27 (1:50, Invitrogen), 20 ng/ml EGF (BD Biosciences), 20 ng/ml bFGF (Miltenyi Biotec) and 4 µg/ml heparin (STEMCELL Technologies), 0.24 µg/ml hydrocortisone (STEMCELL Technologies, and 1% antibiotics. An equal number of cells were cultured to generate spheres, and the number of spheres (diameter > 50 mm) was counted on days 5–7 under a microscope.

To obtain enough cells from the spheroids for the above assays, dissociated cells were cultured at ultra-low dishes (Corning, 3262) for the same period. Spheroids were collected by gentle centrifugation (30×*g*) and dissociated into single cells using 0.05% Trypsin. Cells were then filtered through 70-µm strainers and plated for the next generation of spheres.

### Xenograft model

Four- to six-week-old female NOD/SCID mice were inoculated with 100 µl of cell suspension (1 × 10^6^) in the mammary fat pads under anesthesia via the Matrx VMS anesthesia machine by continuous inhalation of 2% isoflurane gas for 5–10 min. Tumor volume was measured every 3 days after the size was detectable. Tumor volume was calculated by the formula 0.5 × length × width^2^. Mice were euthanized by CO_2_ inhalation 31 days after cell inoculation, and the tumor was measured accurately. Mice were maintained in a pathogen-free (SPF) facility in Biological Resource Centre (BRC), A*STAR. Up to five mice of the same sex were housed in a cage at 20–25 °C and 50% humidity with a 12-h light/dark cycle. The animal study was performed in accordance with animal care and use guidelines approved by the Institutional Animal Care and Use Committee (IACUC; 181412), Agency for Science, Technology and Research (A*STAR), Singapore. The maximum tumor size permitted by IACUC, A*STAR is 2000 mm^3^. The maximal tumor size in this study was not exceeded.

For the metastatic model, 1.25 × 10^6^ WT and NELF-E KO SUM159 cells were injected into 4- to 5-week-old female NGS mice via the tail vein, respectively. Body weight was measured regularly. Mice were maintained in the same condition as stated above. Mice were euthanized by CO_2_ inhalation 34 days after injection, and lung tissues were harvested, inflated with 10% Neutral buffered formalin (NBF), and fixed in the same fixative for further histoprocessing. Sample sectioning, H&E staining, and histopathological evaluation were performed at Advanced Molecular Pathology Laboratory (AMPL), IMCB, A*STAR. Specifically, morphometric analysis (quantitative image analysis) was carried out to quantify the metastatic sites in lung tissues. Microscopic images from mice lungs were captured at 0.7× magnification using a digital camera (DP74, Olympus) fitted on the stereomicroscope (SZX‐16 model, Olympus). CellSense software (Olympus) was used to annotate the area (μm^2^) of each metastatic focus occupied in the pulmonary parenchyma. The area of the entire lung from the given sample was also measured (2–3 sites/animal). The percentage occupancy of metastatic foci was calculated and measured in percentage. The animal study was performed in accordance with animal care and use guidelines approved by the Institutional Animal Care and Use Committee (IACUC; 201572), Agency for Science, Technology and Research (A*STAR), Singapore.

### NELF-E IHC staining

TMA slides were purchased from US Biomax, lnc. IHC staining was performed through Leica Bond III Fully Automated IHC and ISH Staining System with Bond^TM^ Refine Detection Kit (Leica, DS9800) by Advanced Molecular Pathology Laboratory (AMPL), IMCB, A*STAR. Specifically, slides were deparaffinized in Bond^TM^ Dewax Solution and rehydrated through 100% ethanol to 1X Bond^TM^ Wash Solution. Bond^TM^ Epitope Retrieval Solution was used to expose the antigen epitope. After cooling to room temperature, the slides were rinsed with 1X Bond^TM^ Wash solution four times. The slides were incubated in 3–4% (v/v) H_2_O_2_ for 15 min to block endogenous peroxidase and followed by five washes. The slides were then blocked in 10% goat serum for 30 min. NELF-E antibody (Sigma, HPA007187) was added to the slides at the optimized dilution of 1:250 overnight at 4 °C. After rinsing five times in 1X Bond^TM^ Wash Solution, Polymer was added to the slides for 5 min, followed by five washes in 1X Bond^TM^ Wash Solution and another wash in deionized water. Bond^TM^ Mixed DAB Refine was applied for 7 min and then removed by rinsing in deionized water. Nuclei were counterstained with hematoxylin for 5 min and followed by another wash in deionized water and 1X Bond^TM^ wash solution, respectively. In the end, slides were dehydrated and mounted in synthetic mounting media.

### Patient-derived organoid formation assay

The patient-derived organoid culture protocol was adopted from literature^74^ with some modifications. Briefly, organoids were dissociated into smaller clumps of cells and split into an ultra-low attachment 6-well plate (Costar, CLS3471). Organoid cells were then transduced with viruses and incubated at 37 °C overnight. The next day, the virus-transduced cells were collected and resuspended in the 1:1 mixture of cold cultrex growth factor reduced BME type 2 (R&D system, 3533-010-02) and organoid medium^74^, and left to solidify at room temperature for 30 min. Upon completed gelation, an organoid medium containing 1 µg/ml puromycin or blasticidin was added to each well to select stable transduced cells. The stable transduced organoid cells were then dissociated and re-seeded into 24-well suspension plates at the same ratio for each group. The number of organoids was counted on days 3–5 under the microscope. Generation of PDO was done in compliance with all relevant ethical regulations, with informed consent from the patient, and approved by the National Healthcare Group DSRB (reference number: 2015/00357).

### RNA sequencing (RNA-seq)

For this, 1 μg of total RNA was used for RNA-seq library preparation using the NEBNext Ultra II RNA library Prep Kit for Illumina (New England Biolabs, #E770L) following the manufacturer’s instructions. The average fragment length was checked by D1000 ScreenTape (Agilent, 5067–5582) with D1000 reagents (Agilent, 5067–5583). Sample concentration was detected by using the KAPA HiFi HotStart Uracil+ Kit (Roche, KK2801 (07959052001)) or Qubit 3.0 fluorometer (Life technologies). Samples with unique index tags were pooled and sequenced by HiSeq-PE150 (Novogene).

### RNA-seq analysis

Paired-end raw sequencing reads were trimmed with Trim Galore (v0.4.2_dev; https://www.bioinformatics.babraham.ac.uk/projects/ trim_galore/) with parameters: *--trim-n --paired*. Cleaned reads were then mapped to the human hg19 reference genome, guided by the transcript annotations obtained from iGenomes *(*http://igenomes.illumina.com.s3-website-us-east-1.amazonaws.com/Homo_sapiens/UCSC/hg19/Homo_sapiens_UCSC_hg19.tar.gz; accessed on 08/09/2019*)* using the RSEM pipeline (v1.1.11)^75–77^. DeSeq2 (v1.30.1) was applied to differential gene expression analyses with default settings^78^. Genes were considered to be differentially expressed if they showed more than 1.5 or 2-fold difference in expression with an adjusted *p*-value less than 0.05 after correcting for multiple testing by FDR (Benjamini and Hochberg false discovery rate)^79^. MA plots were generated using ggplot2 in R (v4.0.5)^80^.

The single-cell data of ZEB1-overexpressing HMLE cells were obtained from Gene Expression Omnibus (GEO, accession number GSE114397^55^ and analyzed using Seurat (v4.0.6) and Rmagic (v2.0.3) with default parameters^55,81^.

### Gene set enrichment analysis and functional enrichment analysis

We made use of the functions GSEA() and enricher () in the package clusterProfiler^82^ to carry out gene set enrichment analysis (GSEA) and functional enrichment analysis, respectively, against the Molecular Signatures Database (MSigDB)^36,37,83^. *p*-values were calculated based on one million permutations. For both types of analyses, pathways were considered significant if the FDR-corrected *p*-value was ≤0.05.

### Chromatin Immunoprecipitation Sequencing (ChIP-seq)

ChIP-seq was performed as previously described with some modifications^17^. Briefly, cells were dissociated by trypsin and resuspended in fresh media or PBS. For KAT2B ChIP-seq, MCF7ras+SS cells were harvested and double-crosslinked in suspension with 2 mM DSG (disuccinimidyl glutarate, Sigma, 20593) in PBS for 30 min at room temperature, washed with PBS twice, and further crosslinked with formaldehyde (FA) at the final concentration of 1% ((v/v) for 15 min at room temperature. Crosslinking was quenched by the addition of glycine to a final concentration of 125 mM and incubated at room temperature for 15 min. For all other ChIPs, MCF7ras+SS cells were subjected to crosslinking in 1% (v/v) FA for 15 min at room temperature and followed by quenching with 125 mM glycine for 15 min. Cells were then washed twice with ice-cold PBS, and the cell pellets were either snap-frozen for future use or lysed immediately. Lysis buffer 1 (50 mM HEPES (pH 7.5 at 4 °C), 140 mM NaCl, 1 mM EDTA, 10% (v/v) glycerol, 0.5% (v/v) NP-40, and 0.25% (v/v) Triton X-100) with protease inhibitors was added to lyse cells on ice. Released nuclei were centrifuged and washed once with lysis buffer 2 (10 mM Tris (pH 8 at 4 °C), 200 mM NaCl, 1 mM EDTA, and 0.5 mM EGTA) protease inhibitors. Nuclei were then resuspended in lysis buffer 3 (10 mM Tris (pH 7.5 at 4 °C), 140 mM NaCl, 1 mM EDTA, 0.5 mM EGTA, and 0.5% (w/v) N-lauroylsarcosine sodium salt) with protease inhibitors in a Bioruptor-compatible 15-ml tube. Sonication was performed using the Bioruptor Plus (Diagenode, B01020001) for a total of 30 cycles (30 s on; 30 s off) at high power. Insoluble debris were collected by centrifugation (20,000×*g* for 30 min at 4 °C) and discarded. Soluble chromatin was then diluted with an equal volume of weak incubation buffer (50 mM Tris (pH 7.5), 140 mM NaCl, 1 mM EDTA, 0.5 mM EGTA, 1% (v/v) Triton) with protease inhibitors before pre-clearing with Protein A/G Dynabeads (Thermo Fisher Scientific, 1002D/10004D). For ChIP, antibodies were added to 1 mg chromatin for immunoprecipitation at 4 °C overnight with constant rotation. The following day, Dynabeads (protein A+G) were added into samples with constant rotation for 4–6 h. Additional antibody pull-down was performed for SLUG ChIP. The samples were then washed five times with weak incubation buffer with protease inhibitors, followed by LiCl wash (10 mM Tris (pH 7.9), 1 mM EDTA, and 250 mM LiCI). Samples were then washed with TE buffer (10 mM Tris pH 8, 1 mM EDTA) before eluting in ChIP elution buffer (50 mM Tris (pH 8.0), 10 mM EDTA, and 1% (v/v) SDS). Elution was done at 65 °C with constant agitation overnight. After incubating with RNase A (Sigma-Aldrich, R6513) and Proteinase K (Roche, 26709700), reverse-crosslinked DNA was purified with in-house SPRI beads, and sequencing libraries were constructed using the NEBNext Ultra II DNA kit (NEB; catalog number E7645S) following the manufacturer’s protocol. The average fragment length was checked by D1000 ScreenTape (Agilent, 5067–5582) with D1000 reagents (Agilent; Catalog No. 5067–5583), and sample concentration was detected using the Qubit 3.0 fluorometer (Life technologies). Samples with unique index tags were pooled and sequenced by HiSeq-PE150.

### ChIP-seq analysis

Paired-end raw sequencing reads were processed with Trim Galore to trim low-quality reads and remove adapters (with parameters: *--trim-n --paired*). Cleaned reads were then mapped to hg19 reference genome obtained from iGenomes by Bowtie2 (v.2.2.9)^75^ with parameters: *-N 1 -L 25 --no-mixed --no-discordant*. PCR duplicates were removed using SAMtools (v1.4)^84^. Biological replicate alignment files were merged and peaks were called with the MACS2 callpeak function with parameter: --kep-dup all to keep biological duplicate alignments^85^. Peaks with *p*-value ≤10^−9^ were kept for further analysis. ChIPSeeker^86^ was used to annotate peaks to the nearest genes within a window of 10 kb. Bigwig coverage on biological replicates merged BAM files were generated using “bamCompare” command from the package deepTools^87^ with parameters: *-bl hg19-blacklist.v2.bed --ratio subtract -bs 20 -p 16 –normalizeUsing RPKM -b1 IP.bam -b2 input.bam –extendReads*. “hg19-blacklist.v2.bed” was obtained from https://github.com/Boyle-Lab/Blacklist/tree/master/lists/hg19-blacklist.v2.bed.gz. Metaplots were then generated by functions “computeMatrix” and “plotProfile” functions with default parameter settings.

### Peak co-occupancy analysis

Peak co-occupancy analysis was carried out using bedtools package (v2.30.0) by bedtools intersect -u -wa -a factor1.bed -factor2.bed > overlap.bed for overlapping peaks and bedtools intersect -v -wa -a factor1.bed -factor2.bed > factor1_unique.bed for factor-specific peaks. This is relevant to Figs. 5e, f and 6e, f, and Supplementary Figs. 5b, c and 7e.

### Condition-specific peak analysis

To define peaks that are unique to one condition or common to both conditions (for Fig. 5f, g), we adopted the peak co-occupancy analysis on high confidence peaks (*p*-value ≤ 10e-9 called using the MACS2 package) as mentioned above. To confirm there is no global change in SLUG binding before and after NELF-E depletion, we carried out a more quantitative analysis and called condition-specific peaks using bdgdiff() function from MACS2 package with default parameters (relevant to Supplementary Fig. 7e).

### Venn diagram analysis

Venn diagrams presented in this manuscript were generated using Vennerable R package (Jonathan Swinton (2022). Vennerable: Venn and Euler area-proportional diagrams. R package version 3.1.0.9000. (https://github.com/js229/Vennerable).

### Kaplan–Meier plot and correlation analysis

The Kaplan–Meier plotter (https://kmplot.com/analysis/) was used for relapse-free survival (RFS) analysis in breast cancer patients. Patients in all cohorts (*n* = 4929) and the selected cohort of endocrine therapy and chemotherapy (*n* = 510) were both split by the median in the analysis. Pearson’s correlation analysis of *KAT2B* and *ZEB1/2* transcripts in breast cancer patients was performed using data from TCGA (calculation was done by GEPIA, http://gepia.cancer-pku.cn/index.html), METABRIC, and SCAN-B.

### Sample preparation for qPLEX-RIME

Sample preparation protocol for qPLEX-RIME analysis was adapted from literature^44^. In brief, NELF-E pull-down was performed on 50 million MCF7ras+SS cells with and without Dox treatment, and with three biological replicates for each condition. IgG control pull-down was performed similarly for each condition with two biological replicates. MCF7ras+SS cells were harvested and dual crosslinked with 2 mM DSG (Sigma, 20593) for 20 min, followed by incubation in 1% FA for 10 min. Crosslinking was quenched by 0.1 M glycine for 10 min. Nuclei were extracted and chromatin sheared as previously described in the ChIP-seq protocol. Then, 200 μl of Dynabeads® Protein A (Thermo Fisher Scientific, 1002D) and 10 μg of NELF-E antibody (Abcam, ab170104) or Rabbit IgG antibody (Invitrogen, TH275005) were used for each sample. After capturing NELF-E on beads, the beads were washed twice with TEAB buffer (100 mM TEAB, pH 8.5 buffer) and transferred to a new tube. Beads were washed twice more with TEAB buffer and then resuspended with 8 M urea in TEAB buffer. TCEP (Gold Biotechnology, TCEP10) was added to a final concentration of 10 mM and incubated at 25 °C for 30 min. CAA (Sigma-Aldrich, C0267) was then added to a final concentration of 55 mM and incubated in the dark for 30 min. TEAB buffer was added to dilute 8 M urea to below 2 M urea, and 2 µg lysC (Wako, 129-02541) was added for digestion for 4 h at 25 °C. TEAB buffer was then added to dilute urea to below 1 M concentration, and 2.5 µg trypsin (Promega, V511A) was added for digestion for 18 h at 25 °C. Digestion was quenched by the addition of TFA (Sigma-Aldrich, T6508) to a final concentration of 1% (v/v). Peptides were then desalted by solid phase extraction with Oasis HLB 1cc/10 mg cartridges (Waters, 186000383). Cartridges were activated with 300 µl acetonitrile (Merck Supelco, 100029), and equilibrated by passing through 300 µl of water with 0.1 % formic acid (Merck Supelco, 159013) twice. Samples were then loaded, and the cartridge was washed twice with 300 µl of water with 0.1 % formic acid. Peptides were eluted by passing through 250 µl of 80% acetonitrile with 0.1% formic acid in water. Eluted peptides were dried by vacuum centrifugation.

Dried peptides from each condition were resolubilized in 15 µl of TEAB buffer. Then, 5 µl of TMT-10 reagent (Thermo Fisher, 90110) was added to each condition (see Supplementary Fig. 6a and Supplementary Data 3 for TMT-10 labeling channels) and left to incubate at room temperature for 16 h. Labeling was quenched by the addition of 20 µl of 10 mM ammonium formate, pH10 (Sigma-Aldrich, 70221), and samples from each of the TMT-10 channels were pooled together and subjected to high pH fractionation. Spin columns (MoBiTec, M1003) were fitted with a 10-µm pore size filter and loaded with the solid phase, ReproSil Pur Basic resin 10-µm particle size (Dr. Maisch), in acetonitrile. Spin columns were then equilibrated with 100% acetonitrile and conditioned by passing 10 mM ammonium formate pH10 through twice. Samples were loaded and then eluted by step fractionation with 10 mM ammonium formate in increasing acetonitrile concentration (14, 18, 21, 24, 27, 32, 60% ACN). Seven fractions were collected and dried by vacuum centrifugation. Peptides from each fraction were stored at −20 °C before mass spectrometry analysis.

### Liquid chromatography-mass spectrometry and data analysis for qPLEX-RIME

TMT-10 labeled peptides from seven fractions were resupended in water with 2% acetonitrile, 0.5% acetic acid, and 0.06% trifluoroacetic acid and loaded on a heated (50 °C) Easy-Spray 75 µm × 50 cm column on an EASY-nLC 1200 system coupled to a Fusion Mass Spectrometer (Thermo Scientific) with an EASY-Spray source. Peptides were resolved at a flow rate of 400–500 nl/min at 980 bar, with pre-column equilibration by 100% mobile phase A (0.1% formic acid in water) and resolved by increasing mobile phase B (95% acetonitrile in water with 0.1% formic acid) with a gradient as follows: 2–35% B for 70 min, 35–50% B for 10 min, 50–95% B for 5 min, 95% B for 5 min. Mass spectra were collected in Data-Dependent mode with a cycle time of 3 s between master scans. MS1 scans were performed in the Orbitrap with 60K resolution, AGC target of 400,000, and maximum injection time of 100 ms. MS2 scans were collected by Orbitrap with 50K resolution, 42% HCD collision energy, first mass set at 110, AGC target of 80,000, and maximum injection time of 90 ms.

Mass spectra raw files were searched with Mascot in Proteome Discoverer 2.4 against a human Uniprot database (retrieved Jun 2019), with the following parameters: precursor mass tolerance of 10ppm, fragment mass tolerance of 0.06 Da, trypsin as enzyme with maximum three missed cleavages. TMT 10plex was set as a quantification method, with static modifications for carbamidomethyl (C) and dynamic modifications for acetyl (Protein N-terminus), oxidation (M), and deamidation (N,Q). A 1% false discovery rate was set using Percolator node, and Reporter Ions Quantifier nodes were added to the workflow for TMT-10 quantification. Output search files were exported as.txt files and analyzed using an in-house developed R script that utilized the bioconductor *limma* package^88^. TMT-10 intensities across the different conditions were normalized based on total intensity per channel, and the normalized values were used for further analysis. Adjusted *p*-values (adj. *p*-value) were calculated based on Benjamini–Hochberg correction. Interaction networks were made through Cytoscape v3.8.2, with the StringApp used to retrieve and map STRING interaction networks. R script used for the analysis of qPLEX-RIME analysis will be made available upon request.

### Statistical analysis

No statistical methods were used to pre-determine the sample size. The statistical analyses of quantitative reverse-transcription PCR, cell culture experiments, and others were performed using GraphPad Prism (version 9.2.0) and Excel 2016. Statistical tests used in the different experiments were indicated in the respective figure legends.

### Reporting summary

Further information on research design is available in the Nature Portfolio Reporting Summary linked to this article.

## Supplementary information

Supplementary Information

Description of Additional Supplementary Files

Supplementary Data 1

Supplementary Data 2

Supplementary Data 3

Supplementary Data 4

Supplementary Data 5

Supplementary Data 6

Supplementary Data 7

Supplementary Data 8

Reporting Summary

## Data Availability

All raw sequencing data generated in this study have been deposited in the NCBI Gene Expression Omnibus (GEO) under accession number GSE195761. Raw mass spectrometry spectra and search data generated in this study have been uploaded to the jPost repository^89^ with the following accession numbers: JPST001463 (jPOST) and PXD031304 (ProteomeXchange). Single-cell RNA-seq was derived from the published data dataset GSE114397. Molecular Signatures Database (MSigDB) was obtained from https://www.gsea-msigdb.org/gsea/index.jsp. Human genome reference hg19 was obtained from GENCODE [https://www.gencodegenes.org/]. All data are available in the main article, Supplementary Information, and source data. Source data are provided with this paper.

## Source data

Source Data
