# Supplementary material for: Dependency of NELF-E-SLUG-KAT2B epigenetic axis in breast cancer carcinogenesis
Source: Nat Commun. 2023 Apr 28;14:2439. doi: 10.1038/s41467-023-38132-1 (PMC10147683; doi:10.1038/s41467-023-38132-1)
Supplement: Supplementary file 1 — Supplementary Information [file 41467_2023_38132_MOESM1_ESM.pdf]

Supplementary information for

Dependency of NELF-E-SLUG-KAT2B epigenetic axis in breast cancer carcinogenesis

Jieqiong Zhang, Zhenhua Hu, Hwa Hwa Chung, Yun Tian, Kah Weng Lau, Zheng Ser, Yan Ting Lim, Radoslaw M Sobota, Hwei Fen Leong, Benjamin Jieming Chen, Clarisse Jingyi Yeo, Shawn Ying Xuan Tan, Jian Kang, Dennis Eng Kiat Tan, Ieng Fong Sou, Urszula Lucja McClurg, Manikandan Lakshmanan, Thamil Selvan Vaiyapuri, Anandhkumar Raju, Esther Sook Miin Wong, Vinay Tergaonkar, Ravisankar Rajarethinam, Elina Pathak, Wai Leong Tam, Ern Yu Tan and Wee-Wei Tee*

*Correspondence to: Wee-Wei Tee, wwtee@imcb.a-star.edu.sg; Tel: +65 6586 9642; Fax: +65 6586 9642.

This PDF file includes:

Supplementary Figures 1-9

Supplementary Fig. 1

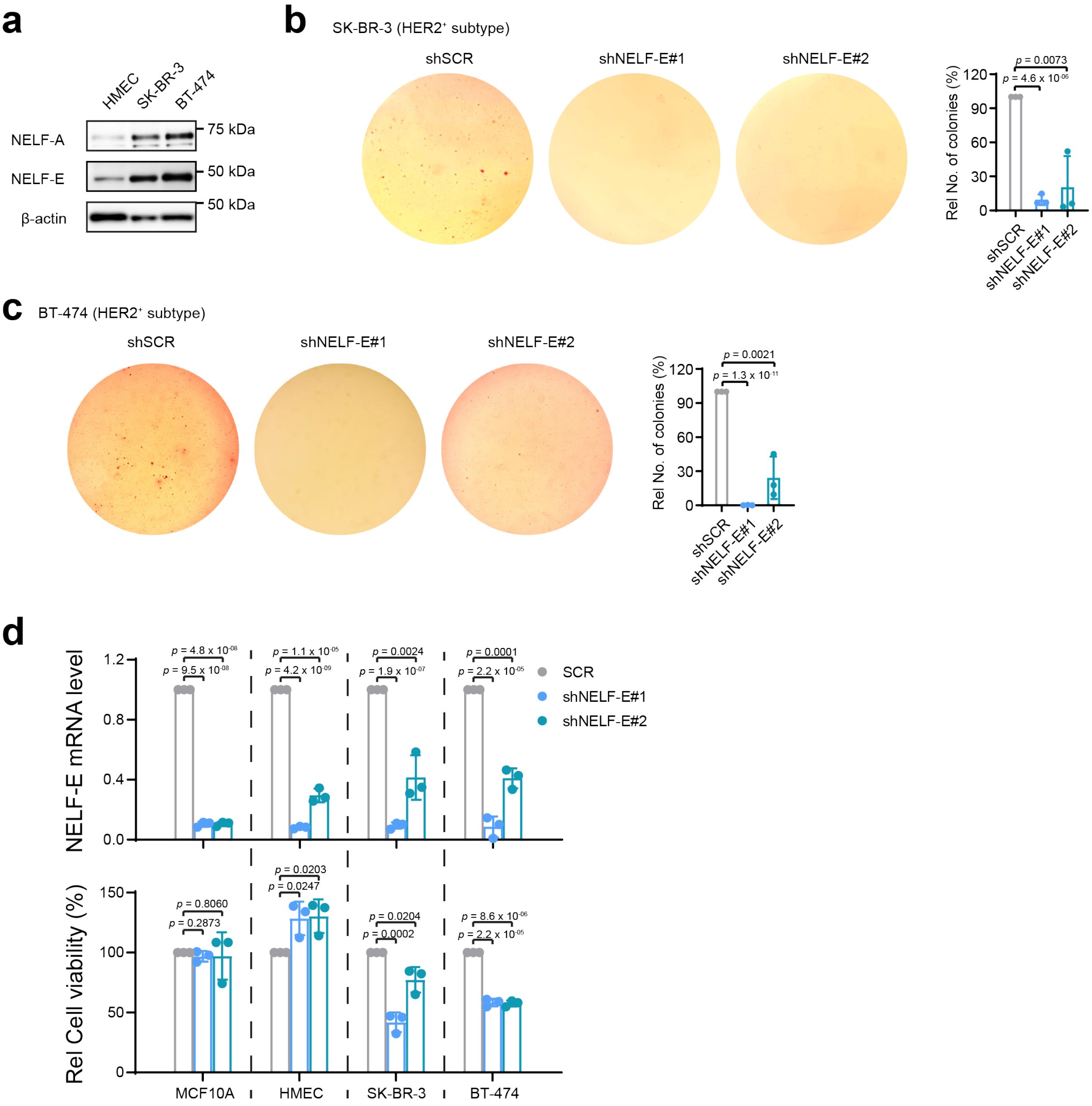

Supplementary Fig. 1 Loss of NELF-E interferes with the proliferation of breast cancer cell lines but not non-tumorigenic breast cell lines.

a Western blot analysis of NELF-A and NELF-E in HMEC, SK-BR-3 and BT-474 cell lines. β-actin was used as the loading control (n = 3). **b,c** Representative images and quantification of the soft agar assays of SK-BR-3 and BT-474 cells transduced with scrambled or two independent shRNAs targeting NELF-E (n = 3). **d** RT-qPCR and cell proliferation analysis of MCF10A, HMEC, SK-BR-3 and BT-474 cells transduced with non-targeting shRNA ('scrambled, shSCR') and two independent shRNAs targeting NELF-E respectively (n = 3). Blots and images are representative of three independent experiments. *P*-values are determined by two-tailed *Student's t-test*. Mean ± SD is represented by bar graphs. Source data are provided as a Source Data file.

Supplementary Fig. 2

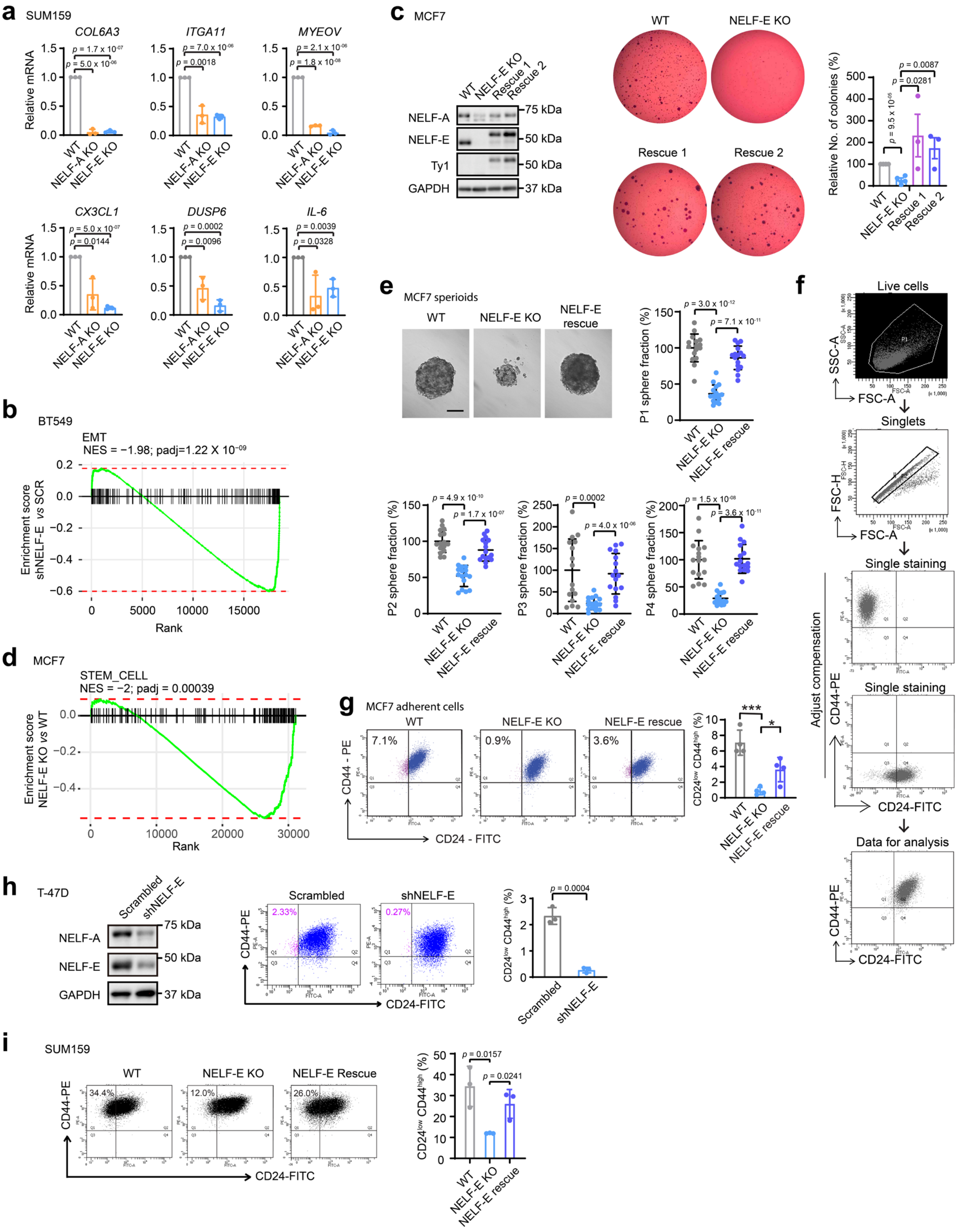

Supplementary Fig. 2 Loss of NELF-E restrains stemness and EMT-related features.

a RT-qPCR analysis of EMT-related genes in SUM159 WT, NELF-A KO and NELF-E KO cells (n = 3). **b** GSEA enrichment plot showing the downregulation of EMT pathway in shNELF-E *vs* SCR BT-549 cells. **c** Left: Western blot analysis of NELF-A, NELF-E and TY1 in WT, NELF-E KO and NELF-E rescue (with 3 x TY1 tag) MCF7 cells. GAPDH was used as the loading control. Middle: Soft agar assays of WT and NELF-E KO MCF7 cells with two NELF-E rescue clones. Right: Colony quantification of the soft agar assays (n = 3). **d** GSEA enrichment plot for mammary stem cell pathway in NELF-E KO MCF7 cells. **e** Left: Mammosphere formation assays in WT, NELF-E KO and NELF-E rescue MCF7 cells (n = 16). Right: Graphs showing quantifications of the spheres at different passages (P1–P4). Scare bar = 200μm. **f** Gating strategy of $CD24^{low}$ $CD44^{high}$ population. **g - i** Flow cytometry analysis and quantification of the $CD24^{low}/CD44^{high}$ population in adherent MCF7 cells (WT, NELF-E KO and NELF-E rescue), T-47D cells (scrambled and NELF-E KD) and SUM159 cells (WT, NELF-E KO and NELF-E rescue) (n = 3).

Blots and images are representative of at least three independent experiments. *P*-values in **a, c, e, g, h, i** are determined by two-tailed *Student's t-test*. Mean ± SD is represented by bar graphs. Source data are provided as a Source Data file.

Supplementary Fig. 3

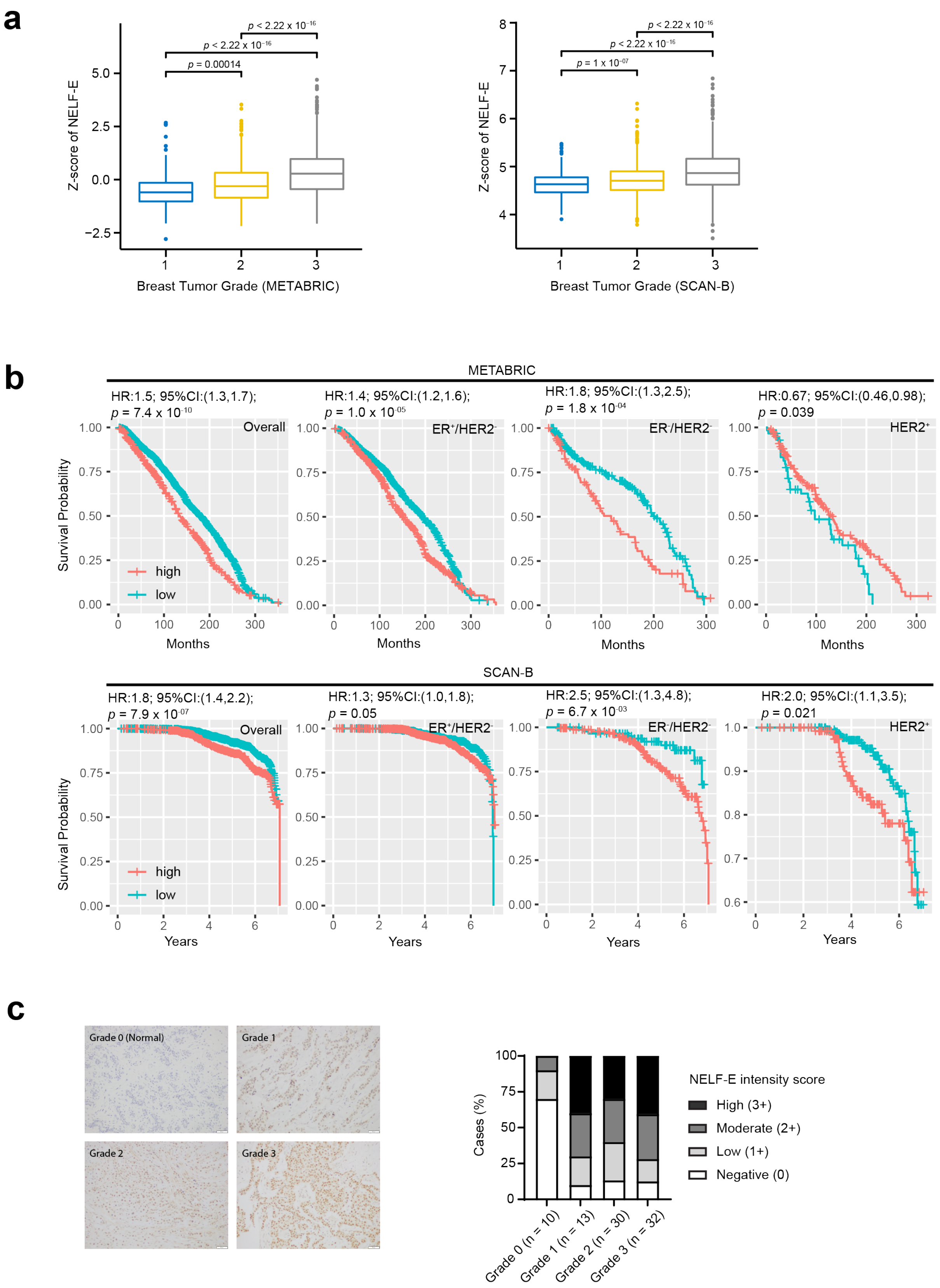

Supplementary Fig. 3 Clinical assessment of NELF-E in breast tumors

a Box plots of normalized NELF-E expression in association with tumor grade in breast tumors from METABRIC and SCAN-B datasets. Centre lines show median values, box limits represent the upper and lower quartiles, and whiskers show 1.5× the interquartile range. *p-values* were determined by two-tailed *Student's t-test*. **b** Patient survival analysis showing that in general, NELF-E expression is negatively associated with overall patient survival. Data was derived from Molecular Taxonomy of Breast Cancer International Consortium (METABRIC) and The Sweden Cancerome Analysis Network – Breast (SCAN-B) datasets respectively. *P*-value was calculated by log-rank test. HR: hazard ratio. **c** IHC staining and scores for NELF-E in normal breast tissue, grade 1, grade 2 and grade 3 breast tumors. Scar bar = 50μm.

Supplementary Fig. 4

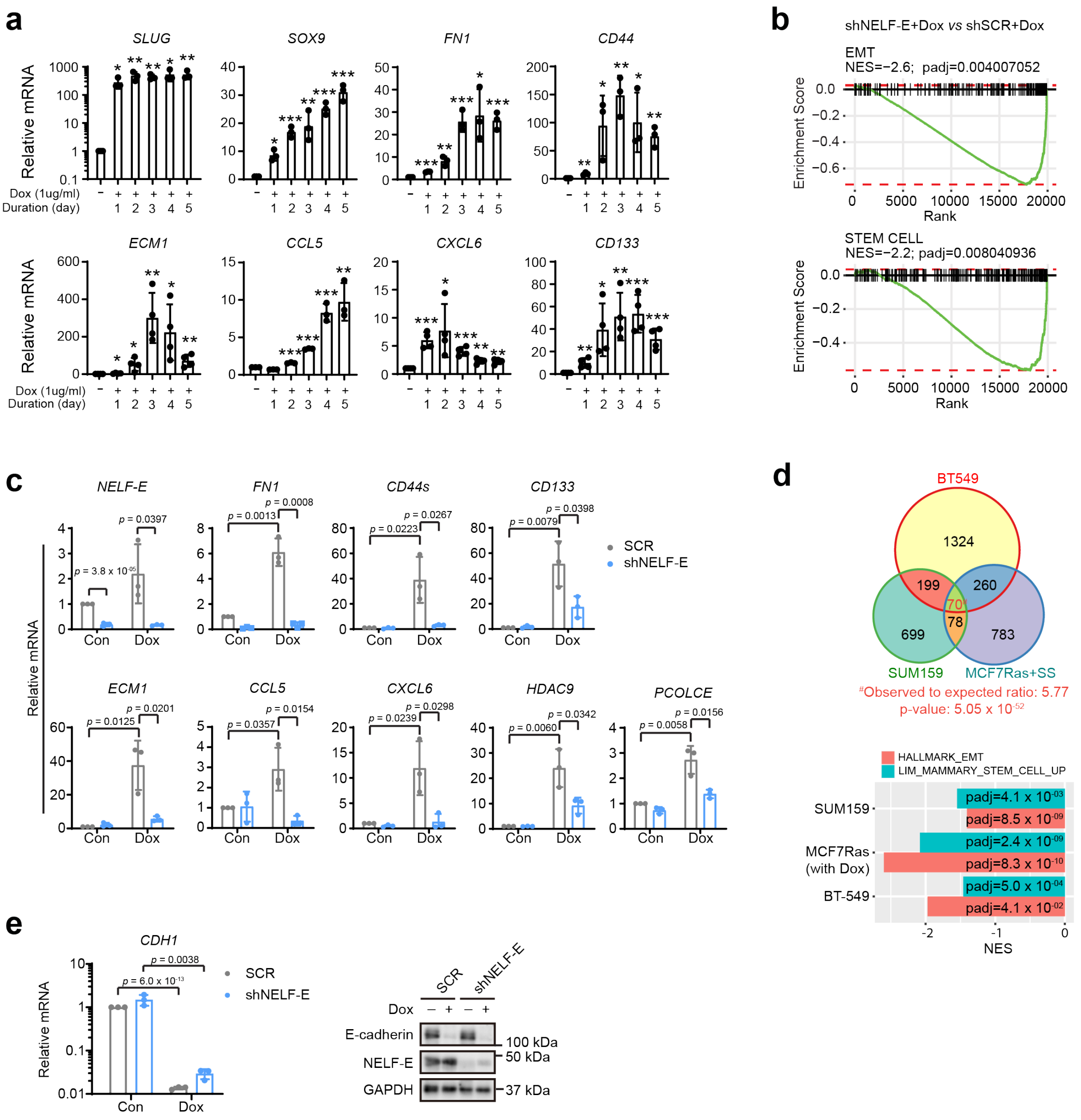

Supplementary Fig. 4 NELF-E KD impairs cancer stemness and EMT pathways

a RT-qPCR analysis of stemness-related and mesenchymal genes in MCF7ras+SS cells treated with Dox for 1-5 days (n = 3 for *SLUG*, *SOX9*, *FN1*, *CD44*, *CCL5*; n = 4 for *ECM1*, *CXCL6*, *CD133*). *, $p < 0.05$; **, $p < 0.01$; ***, $p < 0.001$. Exact *p*-values are provided in Source Data file. **b** GSEA enrichment plots for EMT and stemness-related pathways using RNA-seq results of shNELF-E+Dox *vs* SCR+Dox MCF7ras+SS cells. **c** RT-qPCR analysis of NELF-E, stemness-related and mesenchymal genes in shNELF-E+Dox compared to SCR+Dox MCF7ras+SS cells (n = 3). **d** Top: Venn diagram showing the overlap of downregulated genes in NELF-E KD/KO SUM159 and MCF7ras+SS (with Dox induction) and BT-549 cell lines. [#] Observed to expected ratio and p-value were calculated by package SuperExactTest. Bottom: Standardized GSEA histogram showing normalized enrichment scores of EMT (HALLMARK) and mammary stem cell (LIM) in NELF-E KD/KO *vs* SCR/WT SUM159 and MCF7ras+SS (with Dox induction) and BT-549 cell lines. **e** Left: RT-qPCR analysis of *CDH1* in MCF7ras+SS cells transduced with scrambled or NELF-E shRNA, and as a function of Dox treatment (n = 3). Right: Western blot analysis of E-cadherin and NELF-E in MCF7ras+SS cells (n = 3). GAPDH was used as the loading control.

P-values in **a,c,e** are determined by two-tailed *Student's t-test*. Mean ± SD is represented by bar graphs. Source data are provided as a Source Data file.

Supplementary Fig. 5

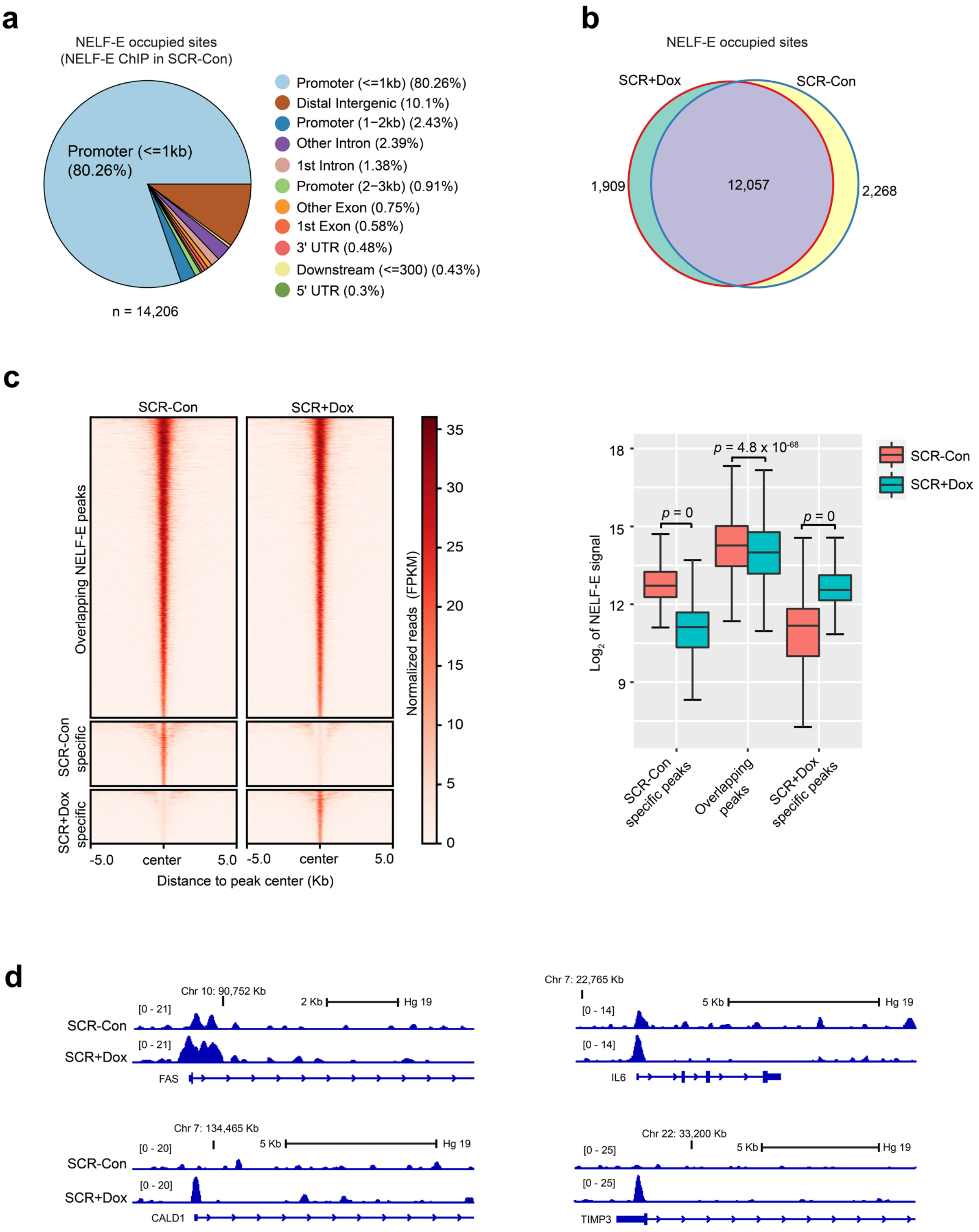

Supplementary Fig. 5 Genome-wide binding profile of NELF-E

a Genomic distribution of NELF-E binding sites in SCR-Con. **b** Venn diagram of NELF-E genomic binding sites in SCR-Con and SCR+Dox cells. **c** Heatmap (left) and boxplot (right) NELF-E binding signals in SCR+Dox and SCR-Con as a function of the NELF-E peak type. *P*-values were calculated from two-tailed Student *t-test*. **d** Genome browser tracks showing the occupancies of NELF-E on *FAS, CALD1, IL6 and TIMP3* loci in MCF7ras+SS cells treated with Dox or vehicle control. Source data are provided as a Source Data file.

Supplementary Fig. 6

a

Channel	Sample
126	IgG_IP_Con_rep1
127N	IgG_IP_Con_rep2
127C	NELFE_IP_Con_rep1
128N	NELFE_IP_Con_rep2
128C	NELFE_IP_Con_rep3
129N	IgG_IP_Dox_rep1
129C	IgG_IP_Dox_rep1
130N	NELFE_IP_Dox_rep1
130C	NELFE_IP_Dox_rep2
131	NELFE_IP_Dox_rep3

b

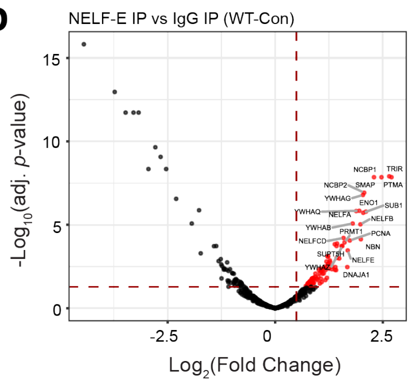

c

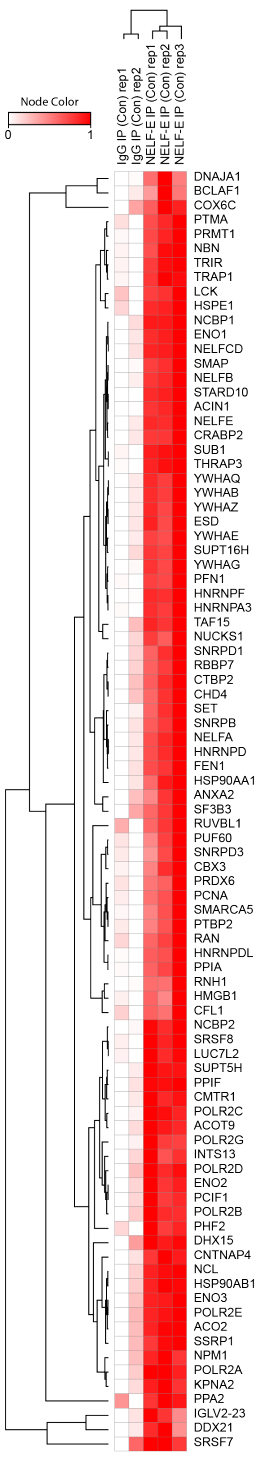

d

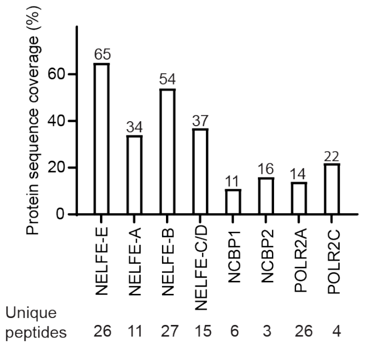

e

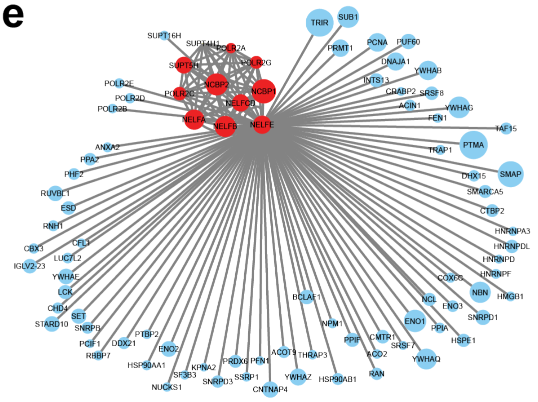

f

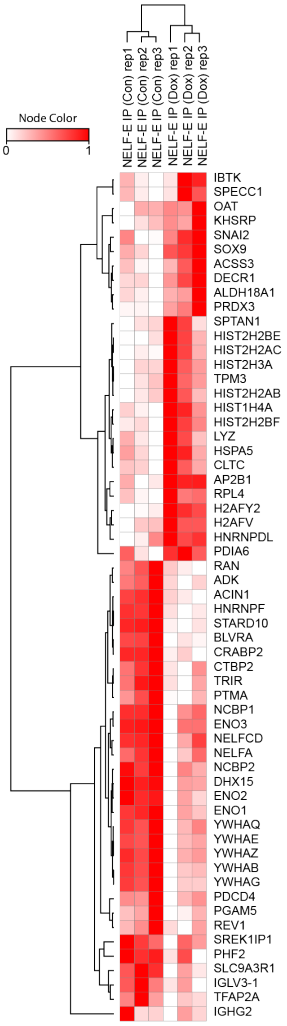

g

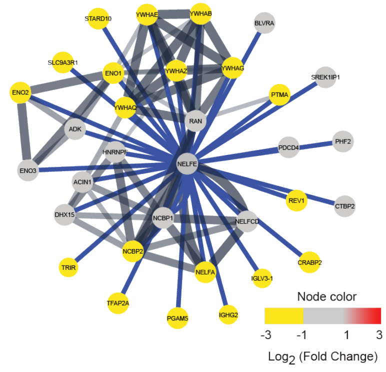

Supplementary Fig. 6 NELF-E qPLEX RIME

a Table depicting TMT-10 channels and labelled conditions for WT-Con and WT+Dox for NELF-E IP and IgG controls. **b** Volcano plot of NELF-E *vs* IgG qPLEX RIME in control MCF7ras+SS cells. Proteins that satisfy the significance threshold of |log2(Fold change)| ≥ 0.5 and adj. *p*-value < 0.05 are colored red and top 20 protein interactors based on fold change are labelled with gene names. *P*-value adjusted by benjamini-hochberg multiple hypothesis correction. **c** Heatmap showing enriched NELF-E interactome over IgG control in WT-Con MCF7ras+SS cells across all replicates. One minus pearson correlation was used for both rows and columns to generate the heatmap. **d** Histogram showing protein sequence coverage (%) and unique peptides of select proteins identified by qPLEX-RIME in MCF7ras+SS cells. **e** Interaction network plot of 89 NELF-E interaction hits (NELF-E IP *vs* IgG controls in WT-Con) as determined by STRING. Interactions detected by qPLEX-RIME are colored in blue, while interactions from the STRING database are colored in red. The circle size of proteins is based on $\log_2$(Fold Change) value. **f** Heatmap showing NELF-E rewired interactome in WT+Dox *vs* WT-Con across all replicates. **g** Interaction network of proteins detected in WT+Dox by qPLEX-RIME and STRING interaction network. Interactions identified from our qPLEX-RIME dataset are colored in blue, while interactions from the STRING database are colored in grey. Proteins are colored based on $\log_2$(fold change) value. Source data are provided as a Source Data file.

Supplementary Fig. 7

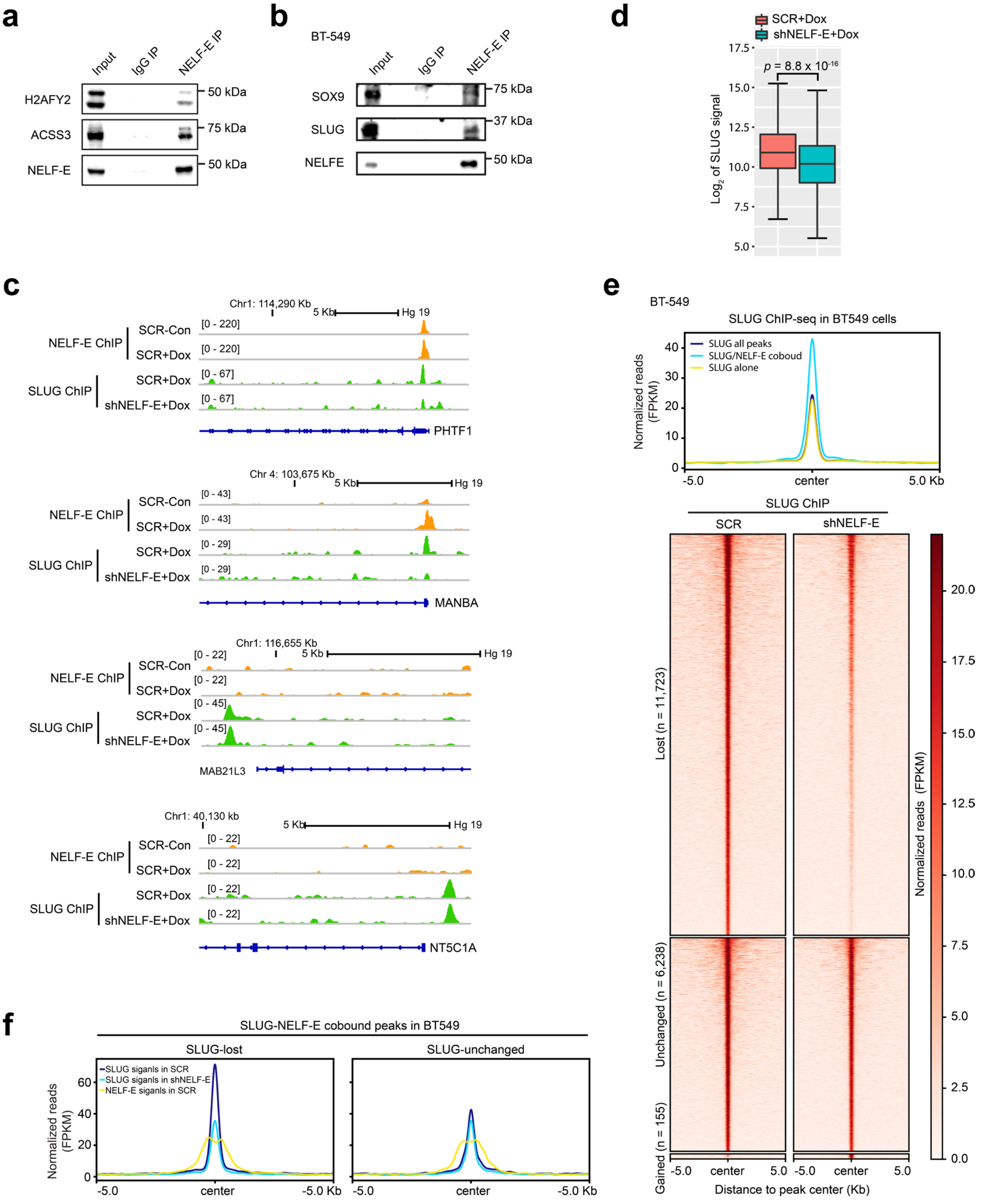

Supplementary Fig. 7 NELF-E regulates SLUG binding

a Western blot analysis of H2AFY2, ACSS3 and NELF-E following endogenous NELF-E IP, using nuclear extracts from Dox-treated MCF7ras+SS cells. **b** Western blot analysis of SOX9, SLUG and NELF-E following endogenous NELF-E IP, using nuclear extracts from parental BT-549 cells. No ectopic expression was performed in the parental BT-549 cells. (**a,b**) Images are representative of three independent experiments. **c** Genome browser tracks showing the occupancies of NELF-E and SLUG on *PHTF1*, *MANBA*, *MAB21L3* and *NT5C1A* loci as examples of NELFE/SLUG-co-bound and SLUG-alone regions. **d** SLUG signals in SCR+Dox and shNELF-E cells across NELF-E *de novo* binding sites in SCR+Dox. *p*-value is calculated from two-tailed *Student's t-test*. **e** Top: Metaplot of SLUG ChIP-seq signal in all, NELF-E/SLUG co-bound and SLUG alone regions. Bottom: Heatmap depiction of SLUG-lost, -gained and -unchanged peaks in SCR *vs* shNELF-E in BT-549 cells. **f** SLUG peaks of high binding strength showing more sensitivity to NELF-E depletion in BT549 cells. Source data are provided as a Source Data file.

Supplementary Fig. 8

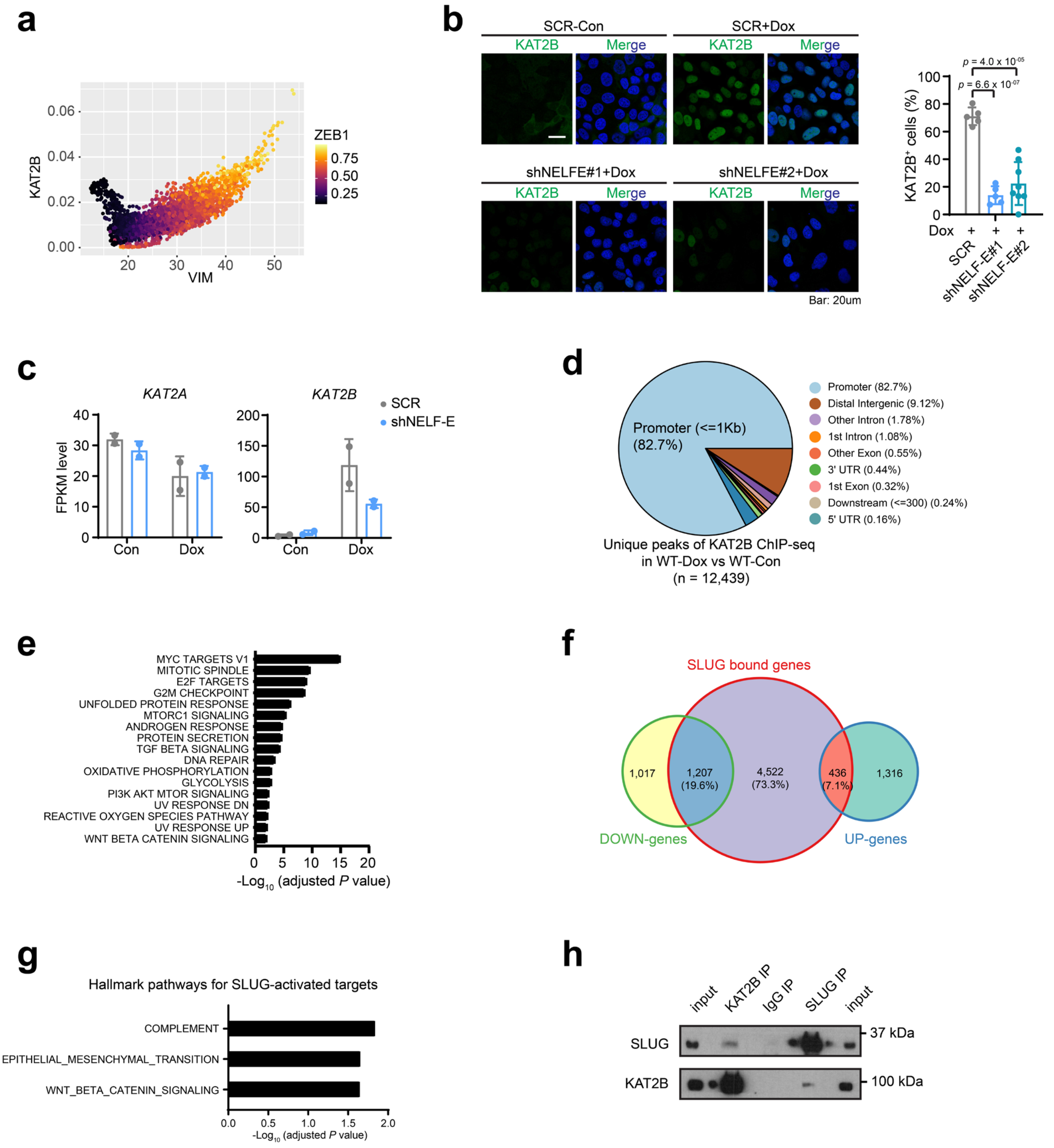

Supplementary Fig. 8 KAT2B positively associates with EMT progression

a Single cell RNA-seq derived from GSE114397 showing that *KAT2B* expression is positively correlated with *ZEB1* and *VIM*. **b** Representative immunofluorescence images and quantification (mean ± SD) of KAT2B-positive MCF7ras+SS cells transduced with scrambled shRNA (n = 5) or two independent NELF-E shRNA #1 and #2 (n = 5 and n = 8 respectively). *P*-values were determined by two-tailed *Student's t-test*. **c** FPKM levels of *KAT2A* and *KAT2B* in MCF7ras+SS cells transduced with scrambled shRNA or two independent NELF-E shRNAs, as a function of Dox treatment. Data is generated from two independent RNA-Seq replicates. **d** Genomic distribution of KAT2B occupied sites (Dox vs vehicle control) in MCF7ras+SS cells. **e** Barplot showing the enrichment of select pathways for KAT2B bound genes upon Dox treatment. *P*-value was calculated from functional enrichment analysis (see details in Methods). **f** Venn diagram showing the overlap between SLUG bound genes and UP- and DOWN-genes. **g** Barplot showing the enrichment of select Hallmark pathways for SLUG-activated genes. *P*-value was calculated from functional enrichment analysis (see details in Methods). **h** Western blot analysis of SLUG and KAT2B following SLUG and KAT2B IP. IP was performed on nuclear extracts from Dox-treated MCF7ras+SS cells. Blots are representative of two independent experiments. Source data are provided as a Source Data file.

Supplementary Fig. 9

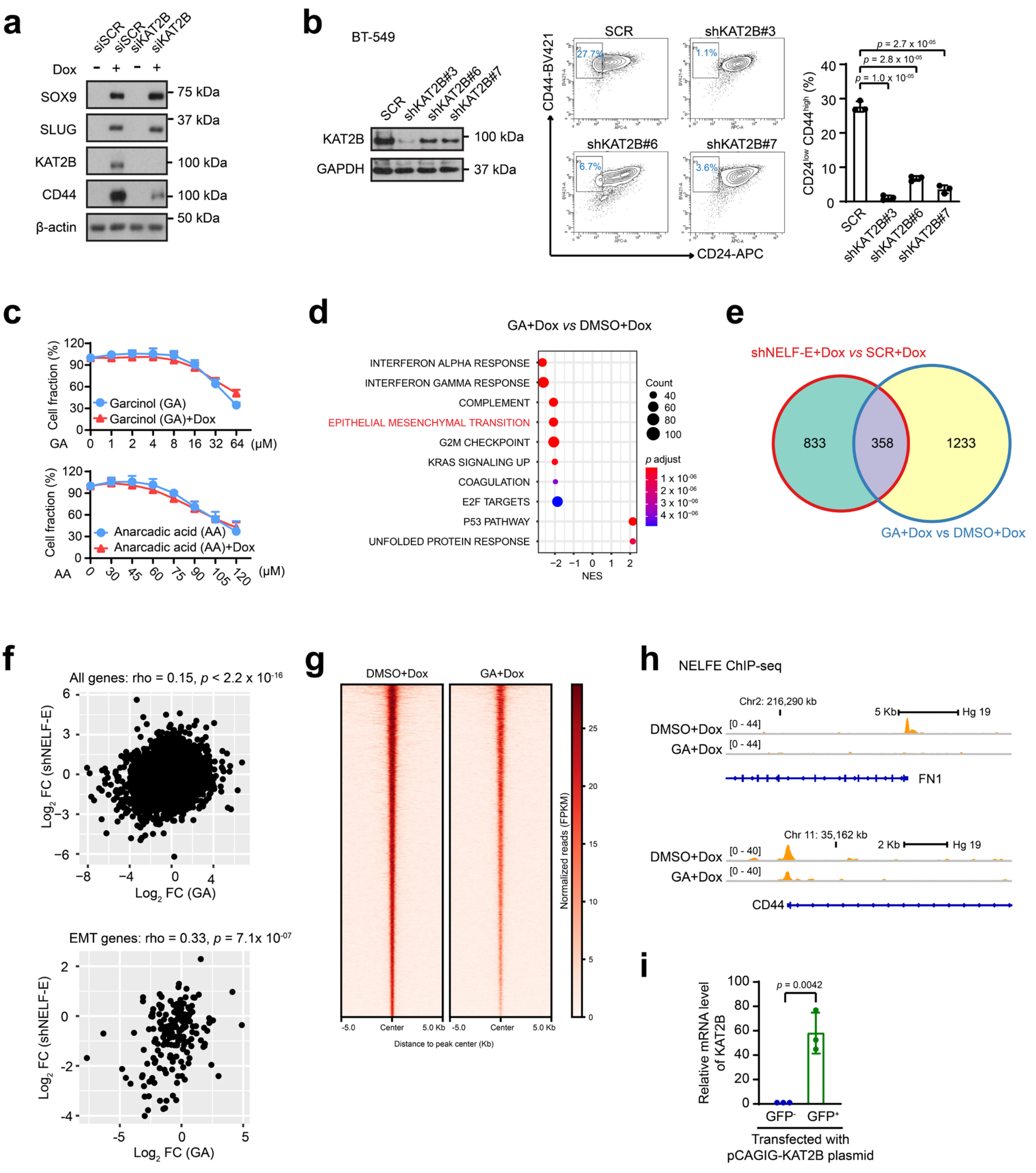

j

Correlation between *KAT2B* and *ZEB1/2* transcripts in patients

	TCGA dataset	METABRICdataset	SCAN-B dataset
Correlation with *ZEB1*	R = 0.36<br>p - value = 0	R = 0.110<br>p - value = 3.8 x 10^{-06}	R = 0.093<br>p - value = 4.3 x 10^{-07}
Correlation with *ZEB2*	R = 0.45<br>p - value = 0	R = 0.120<br>p - value = 4.2 x 10^{-07}	R = 0.350<br>p - value = 6.3 x 10^{-84}

k

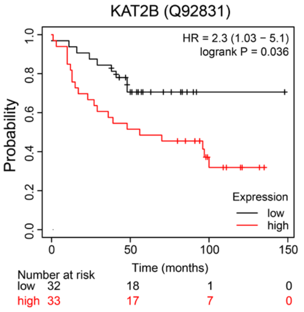

Supplementary Fig. 9 KAT2B promotes EMT progression.

a Western blot analysis of SOX9, SLUG, KAT2B and CD44 in MCF7ras+SS cells treated with scrambled siRNA or KAT2B siRNA, as a function of Dox treatment. GAPDH was used as the loading control. Images are representative of three independent experiments. **b** BT-549 cells were transduced with scrambled shRNA or three independent KAT2B shRNAs, followed by western blot and FACS analysis of the $CD24^{low}$/ $CD44^{high}$ population (n = 3). **c** Cell survival curves showing the proportion of MCF7ras+SS cells treated with different concentrations of GA and AA, as a function of Dox treatment for 72 hours (n = 3). **d** GSEA plot showing the enrichment of select pathways in GA-treated MCF7ras+SS cells compared to vehicle control upon Dox induction. *P*-value was calculated from GSEA (see details in Methods) **e** Venn diagram depicting the overlap between downregulated genes in shNELF-E + Dox vs SCR + Dox, and downregulated genes in GA + Dox cells compared to DMSO + Dox MCF7ras+SS cells. **f** Scatter plots comparing gene transcription changes between shNELF-E vs shSCR and GA-treated cells vs vehicle control-treated cells upon Dox induction. *P*-values were determined by Pearson's test. **g** Heatmap depiction of NELF-E signals in vehicle control and GA-treated MCF7ras+SS cells with Dox induction. **h** Genome browser tracks showing reduced binding of NELF-E on *FN1* and *CD44* promoters in GA-treated cells compared to vehicle control cells. **i** qRT-PCR analysis of *KAT2B* expression in the GFP^{-} and GFP^{+} populations isolated from MCF7ras+SS cells that were transfected with the pCAGIG-KAT2B overexpression plasmid (n = 3). **j** Pearson's correlation analysis of *KAT2B* and *ZEB1/2* transcripts in breast cancer patients. Data was derived from TCGA (calculation was done by GEPIA, http://gepia.cancer-pku.cn/index.html), METABRIC and SCAN-B. **k** Kaplan–Meier analysis showing that KAT2B protein expression is negatively associated with overall patient survival. *P*-value was calculated by a log-rank test. HR: hazard ratio. This analysis was performed by Kaplan-Meier plotter (https://kmplot.com/analysis)

P-values in **b, i** are determined by two-tailed *Student's t-test*. Data in **b,c,i** are presented as Mean ± SD. Source data are provided as a Source Data file.
