# Supplementary material for: Dependency of NELF-E-SLUG-KAT2B epigenetic axis in breast cancer carcinogenesis
Source: Nat Commun. 2023 Apr 28;14:2439. doi: 10.1038/s41467-023-38132-1 (PMC10147683; doi:10.1038/s41467-023-38132-1)
Supplement: Supplementary file 2 — Description of Additional Supplementary Files [file 41467_2023_38132_MOESM2_ESM.pdf]

Description of Additional Supplementary Files

File Name: Supplementary Data 1
Description: List of pathway analysis of NELF-E KO *vs* WT in SUM159
Adjusted *p*-values were calculated by ClusterProfiler package using Benjamini-Hochberg multiple hypothesis correction (see Methods).

File Name: Supplementary Data 2
Differentially expressed genes in NELF-E+Dox vs SCR+Dox MCF7ras+SS cells
Adjusted *p*-values were calculated by DESeq2 package using Benjamini-Hochberg multiple hypothesis correction (see Methods).

File Name: Supplementary Data 3
NELF-E qPLEX RIME data
Two-sided *t-tests* were performed with Benjamini-Hochberg multiple hypothesis correction for the *p*-values.

File Name: Supplementary Data 4
41 genes that are direct targets of NELF-E and SLUG and downregulated in NELF-E KD+Dox vs SCR+Dox

File Name: Supplementary Data 5
List of qPCR primers and sgRNA used in this study

File Name: Supplementary Data 6
List of siRNA used in this study

File Name: Supplementary Data 7
List of antibodies used in this study

File Name: Supplementary Data 8
List of plasmids used in this study
