# Supplementary material for: Dependency of NELF-E-SLUG-KAT2B epigenetic axis in breast cancer carcinogenesis
Source: Nat Commun. 2023 Apr 28;14:2439. doi: 10.1038/s41467-023-38132-1 (PMC10147683; doi:10.1038/s41467-023-38132-1)
Supplement: Supplementary file 11 — Reporting Summary [file 41467_2023_38132_MOESM11_ESM.pdf]

nature portfolio

Corresponding author(s): Wee-Wei Tee

Last updated by author(s): Apr 17, 2023

Reporting Summary

Nature Portfolio wishes to improve the reproducibility of the work that we publish. This form provides structure for consistency and transparency in reporting. For further information on Nature Portfolio policies, see our Editorial Policies and the Editorial Policy Checklist.

Statistics

For all statistical analyses, confirm that the following items are present in the figure legend, table legend, main text, or Methods section.

n/a	Confirmed	
☐	☒	The exact sample size (*n*) for each experimental group/condition, given as a discrete number and unit of measurement
☐	☒	A statement on whether measurements were taken from distinct samples or whether the same sample was measured repeatedly
☐	☒	The statistical test(s) used AND whether they are one- or two-sided<br>*Only common tests should be described solely by name; describe more complex techniques in the Methods section.*
☒	☐	A description of all covariates tested
☐	☒	A description of any assumptions or corrections, such as tests of normality and adjustment for multiple comparisons
☐	☒	A full description of the statistical parameters including central tendency (e.g. means) or other basic estimates (e.g. regression coefficient) AND variation (e.g. standard deviation) or associated estimates of uncertainty (e.g. confidence intervals)
☐	☒	For null hypothesis testing, the test statistic (e.g. *F*, *t*, *r*) with confidence intervals, effect sizes, degrees of freedom and *P* value noted<br>*Give P values as exact values whenever suitable.*
☒	☐	For Bayesian analysis, information on the choice of priors and Markov chain Monte Carlo settings
☐	☒	For hierarchical and complex designs, identification of the appropriate level for tests and full reporting of outcomes
☐	☒	Estimates of effect sizes (e.g. Cohen's *d*, Pearson's *r*), indicating how they were calculated

Our web collection on statistics for biologists contains articles on many of the points above.

Software and code

Policy information about availability of computer code

Data collection	Sequencing samples with unique index tags were pooled and sequenced by illumina HiSeq-PE150 instrument.<br>BD FACSDiva 8.0.2 software was used to collect FACS data.<br>Zen Blue software (v3.3.89.0000) was used to collect microscope images.<br>QuantiStudio Design &Analysis Software (v1.4.3) was used to collect qPCR data.
Data analysis	BD FACSDiva (v8.0.2); QuantiStudio Design &Analysis Software (v1.4.3); Cytoscape (v3.8.2); Image-J 1.53p;Trim Galore (v0.4.2_dev); RSEM(v1.1.11); DESeq2 (v1.30.1) ; Bowtie2(v.2.2.9); SAMtools(v1.4); deeptools (v2.5.3); bedtools (v2.30.0); R (v4.0.5) ; Prism 9 (v9.2.0) and Excel 2016

For manuscripts utilizing custom algorithms or software that are central to the research but not yet described in published literature, software must be made available to editors and reviewers. We strongly encourage code deposition in a community repository (e.g. GitHub). See the Nature Portfolio guidelines for submitting code & software for further information.

Data

Policy information about availability of data

All manuscripts must include a data availability statement. This statement should provide the following information, where applicable:

- Accession codes, unique identifiers, or web links for publicly available datasets
- A description of any restrictions on data availability
- For clinical datasets or third party data, please ensure that the statement adheres to our policy

All raw sequencing data generated in this study have been deposited in the NCBI Gene Expression Omnibus (GEO) under accession number GSE195761 [https://www.ncbi.nlm.nih.gov/geo/query/acc.cgi?acc=GSE195761] . Raw mass spectrometry spectra and search data generated in this study have been uploaded to the jPost repository with the following accession numbers: JPST001463 (jPOST) [https://repository.jpostdb.org/entry/JPST001463.0] and PXD031304 (ProteomeXchange) [https://proteomecentral.proteomexchange.org/cgi/GetDataset?ID=PXD031304]. Single cell RNA-seq was derived from published data dataset GSE114397 [https://www.ncbi.nlm.nih.gov/geo/query/acc.cgi?acc=GSE114397].Molecular Signatures Database (MSigDB) was obtained from https://www.gsea-msigdb.org/gsea/index.jsp. Human genome reference hg19 was obtained from GENCODE [https:// www.gencodegenes.org/]. Source data are provided as a Source Data file.

Human research participants

Policy information about studies involving human research participants and Sex and Gender in Research.

Reporting on sex and gender	Our findings only apply to the female sex. Consent has been taken to share individual-level data for both current and future studies. There was 1 patient in our analysis.
Population characteristics	Our patient was a 53 year-old Chinese female at the time of analysis. She had no comorbidities at the time of diagnosis and was diagnosed with invasive ductal carcinoma in the left breast. She went for a mastectomy before undergoing chemotherapy.
Recruitment	Our patients were screened and recruited by research coordinators in a private room in Tan Tock Seng hospital.
Ethics oversight	National Healthcare Group DSRB approved the study protocol (reference number: 2015/00357)

Note that full information on the approval of the study protocol must also be provided in the manuscript.

Field-specific reporting

Please select the one below that is the best fit for your research. If you are not sure, read the appropriate sections before making your selection.

☒ Life sciences ☐ Behavioural & social sciences ☐ Ecological, evolutionary & environmental sciences

For a reference copy of the document with all sections, see nature.com/documents/nr-reporting-summary-flat.pdf

Life sciences study design

All studies must disclose on these points even when the disclosure is negative.

Sample size	Sample sizes for each experiment are provided in figure legends. The sample sizes were determined based on common practice in the field.
Data exclusions	No data were excluded from analysis
Replication	For ChIP-seq and RNA-seq, experiments were done in two biological replicates. For mouse studies, 4 mice/group were used in fat pad injection and 8 mice/group were used in tail vein injection.<br>Other biological experiments were replicated at least three times.<br>The exact numbers are clearly clarified in the figure legends.
Randomization	Cells were randomly allocated into each group in relevant experiments.
Blinding	The investigators were not blinded to group allocation during data collection and/or analysis as proper controls were already included in the experiment design.

Reporting for specific materials, systems and methods

We require information from authors about some types of materials, experimental systems and methods used in many studies. Here, indicate whether each material, system or method listed is relevant to your study. If you are not sure if a list item applies to your research, read the appropriate section before selecting a response.

Materials & experimental systems

n/a	Involved in the study
☐	☒ Antibodies
☐	☒ Eukaryotic cell lines
☒	☐ Palaeontology and archaeology
☐	☒ Animals and other organisms
☒	☐ Clinical data
☒	☐ Dual use research of concern

Methods

n/a	Involved in the study
☐	☒ ChIP-seq
☐	☒ Flow cytometry
☒	☐ MRI-based neuroimaging

Antibodies

Antibodies used: All antibodies and their dilution used in this study were listed in Supplementary Data 7

Validation: All primary antibodies were validated for western-blot, FACS, IF in human cell lines. Validation details can be found on manufacturer's website.

Eukaryotic cell lines

Policy information about cell lines and Sex and Gender in Research

Cell line source(s):
MCF7 (HTB-22), BT-549 (HTB-122), T-47D (HTB-133) were purchased from ATCC.
SUM159PT (HUMANSUM003006; referred to as SUM159 in the manuscript) was purchased from Bioivt.
MCF7ras+SS was a gift from Dr. Wai Leong Tam (GIS) (PMID 22385965 and 34613780).
SK-BR-3 (HTB-30) and BT-474(HTB-30) were a gift from Dr. Boon Tin Chua and purchased from ATCC.
HMEC (CC-255A) was a gift from Su Chin Tham (IMCB) and purchased from Lonza.

Authentication: MCF7, BT-549, T-47D cells were purchased from ATCC and further verified with short tandem repeat (STR) transcriptomic profiling. SK-BR-3 (HTB-30), BT-474(HTB-30) were purchased from ATCC. HMEC was purchased from Lonza. SUM159 was purchased from Bioivt. MCF7ras+SS is a gift from Dr. Wai Leong Tam (GIS) and validated in previous publications (PMID 22385965 and 34613780).

Mycoplasma contamination: Cells were tested routinely for mycoplasm and are free of contamination at the point of our experiments.

Commonly misidentified lines (See ICLAC register): No commonly misidentified cell lines were used in the study

Animals and other research organisms

Policy information about studies involving animals; ARRIVE guidelines recommended for reporting animal research, and Sex and Gender in Research

Laboratory animals: 4-6 week old NOD/SCID mice for mammary fat pad injection (n = 4) and 4-5 week old NSG mice for tail vein injection (n = 8)

Wild animals: No wild animals were used in this study

Reporting on sex: Only female mice were used in this study to investigate breast cancer.

Field-collected samples: No field collected samples were used in the study

Ethics oversight: The animal study was performed in accordance with animal care and use guidelines approved by the Institutional Animal Care and Use Committee (IACUC; 181412 and 201572), Agency for Science, Technology and Research (A*STAR), Singapore.

Note that full information on the approval of the study protocol must also be provided in the manuscript.

ChIP-seq

Data deposition

☒ Confirm that both raw and final processed data have been deposited in a public database such as GEO.

☒ Confirm that you have deposited or provided access to graph files (e.g. BED files) for the called peaks.

Data access links *May remain private before publication.*: Raw and processed data associated with this study can be viewed under GSE195761

Files in database submission

ChIP-seq.MCF7Ras_KAT2B-ChIP.Con_rep12.bw
ChIP-seq.MCF7Ras_KAT2B-ChIP.DOX_rep12.bw
ChIP-seq.MCF7Ras_NELFE-ChIP.Con_rep12.bw
ChIP-seq.MCF7Ras_NELFE-ChIP.DOX_rep12.bw
ChIP-seq.shNELFE_MCF7Ras_RNAPII-ChIP.scramble_Con_rep12.bw
ChIP-seq.shNELFE_MCF7Ras_RNAPII-ChIP.scramble_DOX_rep12.bw
ChIP-seq.shNELFE_MCF7Ras_RNAPII-ChIP.shNELFE_DOX_rep12.bw
ChIP-seq.shNELFE_MCF7Ras_SLUG-ChIP.scramble_DOX_rep12.bw
ChIP-seq.shNELFE_MCF7Ras_SLUG-ChIP.shNELFE_DOX_rep12.bw
ChIP-seq.MCF7Ras_NELFE-ChIP.DOX_DMSO_rep12.bw
ChIP-seq.MCF7Ras_NELFE-ChIP.DOX_GA_rep12.bw
ChIP-seq.BT549_SLUG-ChIP.scramble_rep12.bw
ChIP-seq.BT549_SLUG-ChIP.shNELFE_rep12.bw
peaks_MCF7Ras.Con_KAT2B_rep12_np09.bed
peaks_MCF7Ras.DOX_KAT2B_rep12_np09.bed
peaks_MCF7Ras.scramble_Con_NELFE_rep12_np09.bed
peaks_MCF7Ras.scramble_DOX_NELFE_rep12_np09.bed
peaks_MCF7Ras.scramble_DOX_SLUG_rep12_np09.bed
peaks_MCF7Ras.shNELFE_DOX_SLUG_rep12_np09.bed
peaks_BT549.scramble_SLUG_rep12_np09.bed
peaks_BT549.scramble_NELFE_rep12_np09.bed
ChIP-seq.MCF7Ras_KAT2B-ChIP.Con_input_rep1_1.fastq.gz
ChIP-seq.MCF7Ras_KAT2B-ChIP.Con_input_rep1_2.fastq.gz
ChIP-seq.MCF7Ras_KAT2B-ChIP.Con_input_rep2_1.fastq.gz
ChIP-seq.MCF7Ras_KAT2B-ChIP.Con_input_rep2_2.fastq.gz
ChIP-seq.MCF7Ras_KAT2B-ChIP.Con_IP_rep1_1.fastq.gz
ChIP-seq.MCF7Ras_KAT2B-ChIP.Con_IP_rep1_2.fastq.gz
ChIP-seq.MCF7Ras_KAT2B-ChIP.Con_IP_rep2_1.fastq.gz
ChIP-seq.MCF7Ras_KAT2B-ChIP.Con_IP_rep2_2.fastq.gz
ChIP-seq.MCF7Ras_KAT2B-ChIP.DOX_input_rep1_1.fastq.gz
ChIP-seq.MCF7Ras_KAT2B-ChIP.DOX_input_rep1_2.fastq.gz
ChIP-seq.MCF7Ras_KAT2B-ChIP.DOX_input_rep2_1.fastq.gz
ChIP-seq.MCF7Ras_KAT2B-ChIP.DOX_input_rep2_2.fastq.gz
ChIP-seq.MCF7Ras_KAT2B-ChIP.DOX_IP_rep1_1.fastq.gz
ChIP-seq.MCF7Ras_KAT2B-ChIP.DOX_IP_rep1_2.fastq.gz
ChIP-seq.MCF7Ras_KAT2B-ChIP.DOX_IP_rep2_1.fastq.gz
ChIP-seq.MCF7Ras_KAT2B-ChIP.DOX_IP_rep2_2.fastq.gz
ChIP-seq.MCF7Ras_NELFE-ChIP.Con_input_rep1_1.fastq.gz
ChIP-seq.MCF7Ras_NELFE-ChIP.Con_input_rep1_2.fastq.gz
ChIP-seq.MCF7Ras_NELFE-ChIP.Con_input_rep2_1.fastq.gz
ChIP-seq.MCF7Ras_NELFE-ChIP.Con_input_rep2_2.fastq.gz
ChIP-seq.MCF7Ras_NELFE-ChIP.Con_IP_rep1_1.fastq.gz
ChIP-seq.MCF7Ras_NELFE-ChIP.Con_IP_rep1_2.fastq.gz
ChIP-seq.MCF7Ras_NELFE-ChIP.Con_IP_rep2_1.fastq.gz
ChIP-seq.MCF7Ras_NELFE-ChIP.Con_IP_rep2_2.fastq.gz
ChIP-seq.MCF7Ras_NELFE-ChIP.DOX_input_rep1_1.fastq.gz
ChIP-seq.MCF7Ras_NELFE-ChIP.DOX_input_rep1_2.fastq.gz
ChIP-seq.MCF7Ras_NELFE-ChIP.DOX_input_rep2_1.fastq.gz
ChIP-seq.MCF7Ras_NELFE-ChIP.DOX_input_rep2_2.fastq.gz
ChIP-seq.MCF7Ras_NELFE-ChIP.DOX_IP_rep1_1.fastq.gz
ChIP-seq.MCF7Ras_NELFE-ChIP.DOX_IP_rep1_2.fastq.gz
ChIP-seq.MCF7Ras_NELFE-ChIP.DOX_IP_rep2_1.fastq.gz
ChIP-seq.MCF7Ras_NELFE-ChIP.DOX_IP_rep2_2.fastq.gz
ChIP-seq.shNELFE_MCF7Ras_RNAPII-ChIP.scramble_Con_input_1.fastq.gz
ChIP-seq.shNELFE_MCF7Ras_RNAPII-ChIP.scramble_Con_input_2.fastq.gz
ChIP-seq.shNELFE_MCF7Ras_RNAPII-ChIP.scramble_Con_IP_rep1_1.fastq.gz
ChIP-seq.shNELFE_MCF7Ras_RNAPII-ChIP.scramble_Con_IP_rep1_2.fastq.gz
ChIP-seq.shNELFE_MCF7Ras_RNAPII-ChIP.scramble_Con_IP_rep2_1.fastq.gz
ChIP-seq.shNELFE_MCF7Ras_RNAPII-ChIP.scramble_Con_IP_rep2_2.fastq.gz
ChIP-seq.shNELFE_MCF7Ras_RNAPII-ChIP.scramble_DOX_input_1.fastq.gz
ChIP-seq.shNELFE_MCF7Ras_RNAPII-ChIP.scramble_DOX_input_2.fastq.gz
ChIP-seq.shNELFE_MCF7Ras_RNAPII-ChIP.scramble_DOX_IP_rep1_1.fastq.gz
ChIP-seq.shNELFE_MCF7Ras_RNAPII-ChIP.scramble_DOX_IP_rep1_2.fastq.gz
ChIP-seq.shNELFE_MCF7Ras_RNAPII-ChIP.scramble_DOX_IP_rep2_1.fastq.gz
ChIP-seq.shNELFE_MCF7Ras_RNAPII-ChIP.scramble_DOX_IP_rep2_2.fastq.gz
ChIP-seq.shNELFE_MCF7Ras_RNAPII-ChIP.shNELFE_DOX_input_1.fastq.gz
ChIP-seq.shNELFE_MCF7Ras_RNAPII-ChIP.shNELFE_DOX_input_2.fastq.gz
ChIP-seq.shNELFE_MCF7Ras_RNAPII-ChIP.shNELFE_DOX_IP_rep1_1.fastq.gz
ChIP-seq.shNELFE_MCF7Ras_RNAPII-ChIP.shNELFE_DOX_IP_rep1_2.fastq.gz
ChIP-seq.shNELFE_MCF7Ras_RNAPII-ChIP.shNELFE_DOX_IP_rep2_1.fastq.gz
ChIP-seq.shNELFE_MCF7Ras_RNAPII-ChIP.shNELFE_DOX_IP_rep2_2.fastq.gz
ChIP-seq.shNELFE_MCF7Ras_SLUG-ChIP.scramble_DOX_input_rep1_1.fastq.gz
ChIP-seq.shNELFE_MCF7Ras_SLUG-ChIP.scramble_DOX_input_rep1_2.fastq.gz
ChIP-seq.shNELFE_MCF7Ras_SLUG-ChIP.scramble_DOX_input_rep2_1.fastq.gz
ChIP-seq.shNELFE_MCF7Ras_SLUG-ChIP.scramble_DOX_input_rep2_2.fastq.gz

ChIP-seq.shNELFE_MCF7Ras_SLUG-ChIP.scramble_DOX_IP_rep1_1.fastq.gz
ChIP-seq.shNELFE_MCF7Ras_SLUG-ChIP.scramble_DOX_IP_rep1_2.fastq.gz
ChIP-seq.shNELFE_MCF7Ras_SLUG-ChIP.scramble_DOX_IP_rep2_1.fastq.gz
ChIP-seq.shNELFE_MCF7Ras_SLUG-ChIP.scramble_DOX_IP_rep2_2.fastq.gz
ChIP-seq.shNELFE_MCF7Ras_SLUG-ChIP.shNELFE_DOX_input_rep1_1.fastq.gz
ChIP-seq.shNELFE_MCF7Ras_SLUG-ChIP.shNELFE_DOX_input_rep1_2.fastq.gz
ChIP-seq.shNELFE_MCF7Ras_SLUG-ChIP.shNELFE_DOX_input_rep2_1.fastq.gz
ChIP-seq.shNELFE_MCF7Ras_SLUG-ChIP.shNELFE_DOX_input_rep2_2.fastq.gz
ChIP-seq.shNELFE_MCF7Ras_SLUG-ChIP.shNELFE_DOX_IP_rep1_1.fastq.gz
ChIP-seq.shNELFE_MCF7Ras_SLUG-ChIP.shNELFE_DOX_IP_rep1_2.fastq.gz
ChIP-seq.shNELFE_MCF7Ras_SLUG-ChIP.shNELFE_DOX_IP_rep2_1.fastq.gz
ChIP-seq.shNELFE_MCF7Ras_SLUG-ChIP.shNELFE_DOX_IP_rep2_2.fastq.gz
ChIP-seq.MCF7Ras_NELFE-ChIP.DOX_DMSO_IP_rep1_1.fastq.gz
ChIP-seq.MCF7Ras_NELFE-ChIP.DOX_DMSO_IP_rep1_2.fastq.gz
ChIP-seq.MCF7Ras_NELFE-ChIP.DOX_DMSO_IP_rep2_1.fastq.gz
ChIP-seq.MCF7Ras_NELFE-ChIP.DOX_DMSO_IP_rep2_2.fastq.gz
ChIP-seq.MCF7Ras_NELFE-ChIP.DOX_GA_IP_rep1_1.fastq.gz
ChIP-seq.MCF7Ras_NELFE-ChIP.DOX_GA_IP_rep1_2.fastq.gz
ChIP-seq.MCF7Ras_NELFE-ChIP.DOX_GA_IP_rep2_1.fastq.gz
ChIP-seq.MCF7Ras_NELFE-ChIP.DOX_GA_IP_rep2_2.fastq.gz
ChIP-seq.MCF7Ras_NELFE-ChIP.DOX_DMSO_input_1.fastq.gz
ChIP-seq.MCF7Ras_NELFE-ChIP.DOX_DMSO_input_2.fastq.gz
ChIP-seq.MCF7Ras_NELFE-ChIP.DOX_GA_input_1.fastq.gz
ChIP-seq.MCF7Ras_NELFE-ChIP.DOX_GA_input_2.fastq.gz
ChIP-seq.BT549_SLUG-ChIP.scramble_IP_rep1_1.fastq.gz
ChIP-seq.BT549_SLUG-ChIP.scramble_IP_rep1_2.fastq.gz
ChIP-seq.BT549_SLUG-ChIP.scramble_IP_rep2_1.fastq.gz
ChIP-seq.BT549_SLUG-ChIP.scramble_IP_rep2_2.fastq.gz
ChIP-seq.BT549_SLUG-ChIP.shNELFE_IP_rep1_1.fastq.gz
ChIP-seq.BT549_SLUG-ChIP.shNELFE_IP_rep1_2.fastq.gz
ChIP-seq.BT549_SLUG-ChIP.shNELFE_IP_rep2_1.fastq.gz
ChIP-seq.BT549_SLUG-ChIP.shNELFE_IP_rep2_2.fastq.gz
ChIP-seq.BT549_SLUG-ChIP.scramble_input_1.fastq.gz
ChIP-seq.BT549_SLUG-ChIP.scramble_input_2.fastq.gz
ChIP-seq.BT549_SLUG-ChIP.shNELFE_input_1.fastq.gz
ChIP-seq.BT549_SLUG-ChIP.shNELFE_input_2.fastq.gz
ChIP-seq.BT549_NELFE-ChIP.scramble_IP_rep1_1.fastq.gz
ChIP-seq.BT549_NELFE-ChIP.scramble_IP_rep1_2.fastq.gz
ChIP-seq.BT549_NELFE-ChIP.scramble_IP_rep2_1.fastq.gz
ChIP-seq.BT549_NELFE-ChIP.scramble_IP_rep2_2.fastq.gz
ChIP-seq.BT549_NELFE-ChIP.scramble_input_rep1_1.fastq.gz
ChIP-seq.BT549_NELFE-ChIP.scramble_input_rep1_2.fastq.gz
ChIP-seq.BT549_NELFE-ChIP.scramble_input_rep2_1.fastq.gz
ChIP-seq.BT549_NELFE-ChIP.scramble_input_rep2_2.fastq.gz

Genome browser session
(e.g. UCSC)

N/A

Methodology

Replicates

All ChIP-seq experiments are done in two biological replicates.

Sequencing depth

sample total reads unique reads read length type
ChIP-seq.MCF7Ras_KAT2B-ChIP.Con_input_rep1 58,306,702 48,525,032 150bp Paired-end
ChIP-seq.MCF7Ras_KAT2B-ChIP.Con_input_rep2 74,908,778 62,466,304 150bp Paired-end
ChIP-seq.MCF7Ras_KAT2B-ChIP.Con_IP_rep1 59,835,342 49,185,093 150bp Paired-end
ChIP-seq.MCF7Ras_KAT2B-ChIP.Con_IP_rep2 69,664,014 57,627,154 150bp Paired-end
ChIP-seq.MCF7Ras_KAT2B-ChIP.DOX_input_rep1 55,060,075 45,702,761 150bp Paired-end
ChIP-seq.MCF7Ras_KAT2B-ChIP.DOX_input_rep2 63,465,460 52,705,459 150bp Paired-end
ChIP-seq.MCF7Ras_KAT2B-ChIP.DOX_IP_rep1 57,065,432 46,411,841 150bp Paired-end
ChIP-seq.MCF7Ras_KAT2B-ChIP.DOX_IP_rep2 57,820,590 46,883,515 150bp Paired-end
ChIP-seq.MCF7Ras_NELFE-ChIP.DOX_input_rep1 63,953,614 53,944,403 150bp Paired-end
ChIP-seq.MCF7Ras_NELFE-ChIP.DOX_input_rep2 39,434,791 32,509,889 150bp Paired-end
ChIP-seq.MCF7Ras_NELFE-ChIP.DOX_IP_rep1 72,113,168 59,482,715 150bp Paired-end
ChIP-seq.MCF7Ras_NELFE-ChIP.DOX_IP_rep2 69,813,225 57,950,549 150bp Paired-end
ChIP-seq.MCF7Ras_NELFE-ChIP.noDOX_input_rep1 59,310,209 49,329,195 150bp Paired-end
ChIP-seq.MCF7Ras_NELFE-ChIP.noDOX_input_rep2 41,522,518 34,297,333 150bp Paired-end
ChIP-seq.MCF7Ras_NELFE-ChIP.noDOX_IP_rep1 72,500,807 61,112,113 150bp Paired-end
ChIP-seq.MCF7Ras_NELFE-ChIP.noDOX_IP_rep2 52,657,407 43,733,032 150bp Paired-end
ChIP-seq.shNELFE_MCF7Ras_RNAPII-ChIP.scramble_Con_input 117,012,699 97,897,013 150bp Paired-end
ChIP-seq.shNELFE_MCF7Ras_RNAPII-ChIP.scramble_Con_IP_rep1 92,004,573 77,084,542 150bp Paired-end
ChIP-seq.shNELFE_MCF7Ras_RNAPII-ChIP.scramble_Con_IP_rep2 97,261,859 80,247,642 150bp Paired-end
ChIP-seq.shNELFE_MCF7Ras_RNAPII-ChIP.scramble_DOX_input 102,701,406 85,211,306 150bp Paired-end
ChIP-seq.shNELFE_MCF7Ras_RNAPII-ChIP.scramble_DOX_IP_rep1 80,602,883 66,616,498 150bp Paired-end
ChIP-seq.shNELFE_MCF7Ras_RNAPII-ChIP.scramble_DOX_IP_rep2 76,111,882 62,598,164 150bp Paired-end
ChIP-seq.shNELFE_MCF7Ras_RNAPII-ChIP.shNELFE_DOX_input 110,061,631 91,926,728 150bp Paired-end

ChIP-seq.shNELFE_MCF7Ras_RNAPII-ChIP.shNELFE_DOX_IP_rep1 76,559,026 62,806,835 150bp Paired-end
ChIP-seq.shNELFE_MCF7Ras_RNAPII-ChIP.shNELFE_DOX_IP_rep2 82,568,322 68,001,165 150bp Paired-end
ChIP-seq.shNELFE_MCF7Ras_SLUG-ChIP.scramble_DOX_input_rep2 54,200,505 44,826,746 150bp Paired-end
ChIP-seq.shNELFE_MCF7Ras_SLUG-ChIP.scramble_DOX_IP_rep1 42,066,053 34,175,445 150bp Paired-end
ChIP-seq.shNELFE_MCF7Ras_SLUG-ChIP.scramble_DOX_IP_rep2 93,968,793 78,456,423 150bp Paired-end
ChIP-seq.shNELFE_MCF7Ras_SLUG-ChIP.shNELFE_DOX_input_rep 50,196,160 50,196,160 150bp Paired-end
ChIP-seq.shNELFE_MCF7Ras_SLUG-ChIP.shNELFE_DOX_IP_rep1 47,522,673 38,758,736 150bp Paired-end
ChIP-seq.shNELFE_MCF7Ras_SLUG-ChIP.shNELFE_DOX_IP_rep2 82,758,465 68,776,540 150bp Paired-end
ChIP-seq.MCF7Ras_NELFE-ChIP.DOX_DMSO_IP_rep1 43,655,103 35,302,317 150bp Paired-end
ChIP-seq.MCF7Ras_NELFE-ChIP.DOX_DMSO_IP_rep1 48,578,558 38,684,347 150bp Paired-end
ChIP-seq.MCF7Ras_NELFE-ChIP.DOX_GA_IP_rep1 53,437,840 43,882,297 150bp Paired-end
ChIP-seq.MCF7Ras_NELFE-ChIP.DOX_GA_IP_rep2 53,941,200 43,127,526 150bp Paired-end
ChIP-seq.MCF7Ras_NELFE-ChIP.DOX_DMSO_input 58,668,397 47,943,608 150bp Paired-end
ChIP-seq.MCF7Ras_NELFE-ChIP.DOX_GA_input 73,695,893 60,977,051 150bp Paired-end
ChIP-seq.BT549_SLUG-ChIP.scramble_IP_rep1 86,671,796 69,959,939 150bp Paired-end
ChIP-seq.BT549_SLUG-ChIP.scramble_IP_rep2 84,893,680 69,520,801 150bp Paired-end
ChIP-seq.BT549_SLUG-ChIP.shNELFE_IP_rep1 80,622,868 66,090,570 150bp Paired-end
ChIP-seq.BT549_SLUG-ChIP.shNELFE_IP_rep2 85,967,967 70,983,405 150bp Paired-end
ChIP-seq.BT549_SLUG-ChIP.scramble_input 92,881,228 76,702,711 150bp Paired-end
ChIP-seq.BT549_SLUG-ChIP.shNELFE_input 99,070,002 81,558,910 150bp Paired-end
ChIP-seq.BT549_NELFE-ChIP.scramble_IP_rep1 39,773,320 32,937,840 150bp Paired-end
ChIP-seq.BT549_NELFE-ChIP.scramble_IP_rep2 34,212,611 27,768,792 150bp Paired-end
ChIP-seq.BT549_NELFE-ChIP.scramble_input_rep1 37553723 30662809 150bp Paired-end
ChIP-seq.BT549_NELFE-ChIP.scramble_input_rep2 38,563,240 31,445,192 150bp Paired-end

Antibodies

Anti-NELF-E, Abcam, ab170104
KAT2B, Cell Signaling Technology, 3378S
SLUG, Cell Signaling Technology, 9585S
RNA polymerase II (clone CTD4H8), Millipore,05-623
Details of antibodies and their amount in ChIP-seq are provided in Supplementary Data 7.

Peak calling parameters

macs2 callpeak -t IP.rmdup.bam -c INPUT.rmdup.bam -n IP_name -g hs -f BAMPE --keep-dup all. "keep-dup all" is used because the PCR duplicates have already been removed before merging.

Data quality

Only peaks of high quality were kept by applying the p <= 10e-09. In addition, Peaks that overlap blacklist regions were removed.

Software

Trim Galore (v0.4.2_dev); Bowtie2(v.2.2.9); SAMtools(v1.4); deeptools (v2.5.3); macs2(v2.1.1); bedtools (v2.30.0)

Flow Cytometry

Plots

Confirm that:

☒ The axis labels state the marker and fluorochrome used (e.g. CD4-FITC).

☒ The axis scales are clearly visible. Include numbers along axes only for bottom left plot of group (a 'group' is an analysis of identical markers).

☒ All plots are contour plots with outliers or pseudocolor plots.

☒ A numerical value for number of cells or percentage (with statistics) is provided.

Methodology

Sample preparation

MCF7, T47D, BT549, SUM159 and MCF7ras+SS cells were dislodged with trypsin and incubated with FACS blocking buffer (1X PBS, 3% BSA) for 30 minutes and then stained with antibodies listed in the supplementary table for 45 minutes.
After washing three times with PBS, resuspended cells were passed through a cell strainer (0.7 um) into FACS tube. Samples were transported on ice to FACS facility.

Instrument

FACS analysis was analyzed with the LSRII machine (BD).

Software

The standard issued manufacturer's software was used without any further modifications. The routine updates/maintenance and export of FACS data was handled by the FACS facility within A*STAR. BD FACSDiva 8.0.2 software was used for data collection and analysis.

Cell population abundance

10,000-30,000 live cells were selected for singlets and following CD24 CD44 population analysis. CD24low CD44high populations (%) were clarified in the respective figures.

Gating strategy

Gating strategy was described in Supplementary Fig. 2f

☒ Tick this box to confirm that a figure exemplifying the gating strategy is provided in the Supplementary Information.
